# Identification of regulatory networks and crosstalk factors in brown adipose tissue and liver of a cold-exposed cardiometabolic mouse model

**DOI:** 10.1186/s12933-024-02397-7

**Published:** 2024-08-14

**Authors:** Melina Amor, Malena Diaz, Valentina Bianco, Monika Svecla, Birgit Schwarz, Silvia Rainer, Anita Pirchheim, Laszlo Schooltink, Suravi Mukherjee, Gernot F. Grabner, Giangiacomo Beretta, Claudia Lamina, Giuseppe Danilo Norata, Hubert Hackl, Dagmar Kratky

**Affiliations:** 1 Gottfried Schatz Research Center, Molecular Biology and Biochemistry, Medical University of Graz, Neue Stiftingtalstrasse 6/4, Graz, 8010 Austria; 2 Department of Pharmacological and Biomolecular Sciences, University of Milan, Milan, Italy; 3 Department of Environmental Science and Policy, Università degli Studi di Milano, Milan, Italy; 4 Department of Genetics and Pharmacology, Institute of Genetic Epidemiology, Medical University of Innsbruck, Innsbruck, Austria; 5 Institute of Bioinformatics, Medical University of Innsbruck, Innsbruck, Austria; 6 BioTechMed-Graz, Graz, Austria; 7 Present Address: Department of Medicine and Surgery, University of Parma, Parma, Italy; 8 Present Address: Department of Neurosurgery, Charité– Universitätsmedizin Berlin, Berlin, Germany

**Keywords:** Brown adipose tissue, Liver, Cardiometabolic diseases, Cold exposure, Ldlr-deficient mice, Untargeted proteomics

## Abstract

**Background:**

Activation of brown adipose tissue (BAT) has gained attention due to its ability to dissipate energy and counteract cardiometabolic diseases (CMDs).

**Methods:**

This study investigated the consequences of cold exposure on the BAT and liver proteomes of an established CMD mouse model based on LDL receptor-deficient (LdlrKO) mice fed a high-fat, high-sucrose, high-cholesterol diet for 16 weeks. We analyzed energy metabolism in vivo and performed untargeted proteomics on BAT and liver of LdlrKO mice maintained at 22 °C or 5 °C for 7 days.

**Results:**

We identified several dysregulated pathways, miRNAs, and transcription factors in BAT and liver of cold-exposed Ldlrko mice that have not been previously described in this context. Networks of regulatory interactions based on shared downstream targets and analysis of ligand-receptor pairs identified fibrinogen alpha chain (FGA) and fibronectin 1 (FN1) as potential crosstalk factors between BAT and liver in response to cold exposure. Importantly, genetic variations in the genes encoding *FGA* and *FN1* have been associated with cardiometabolic-related phenotypes and traits in humans.

**Discussion:**

This study describes the key factors, pathways, and regulatory networks involved in the crosstalk between BAT and the liver in a cold-exposed CMD mouse model. These findings may provide a basis for future studies aimed at testing whether molecular mediators, as well as regulatory and signaling mechanisms involved in tissue adaption upon cold exposure, could represent a target in cardiometabolic disorders.

**Graphical abstract:**

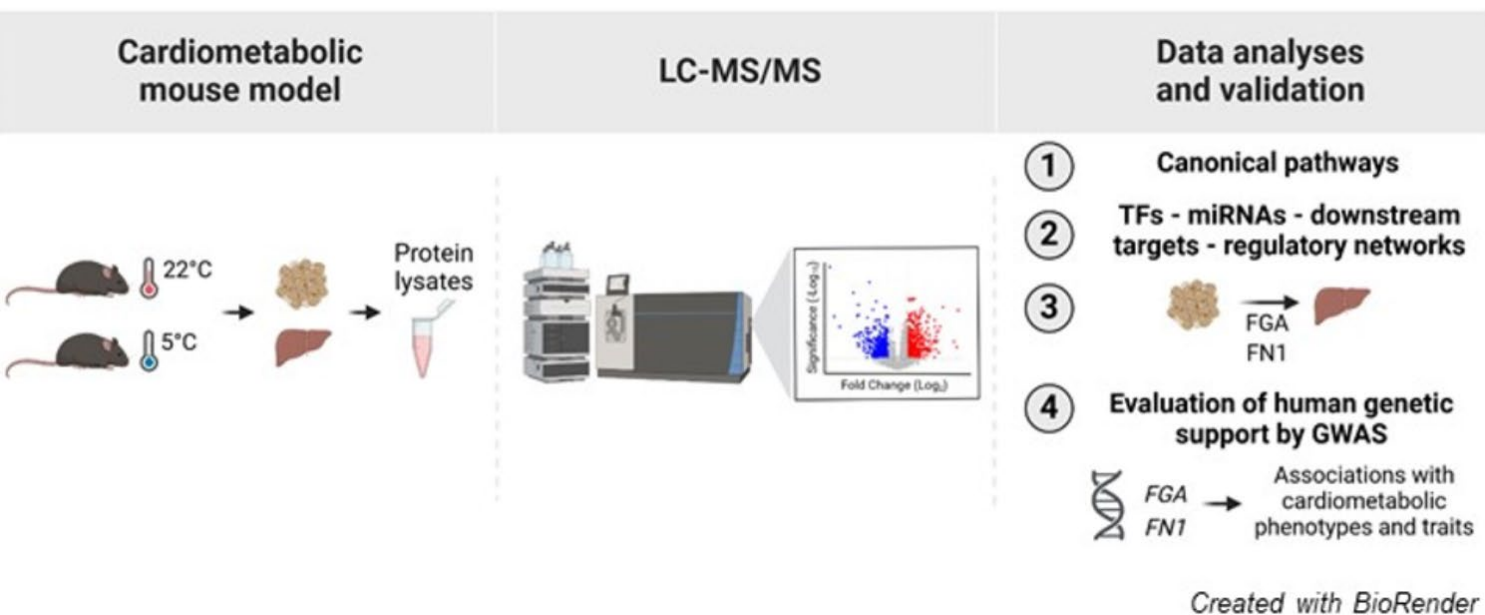

**Supplementary Information:**

The online version contains supplementary material available at 10.1186/s12933-024-02397-7.

## Background

Cardiometabolic diseases (CMDs) are a global health concern, as they are the leading cause of death in Western societies and are also on the rise in low- and middle-income countries [1]. The increasing prevalence of CMDs is a direct consequence of the global obesity pandemic and requires immediate attention and intervention. Although lifestyle and dietary modifications should be the primary consideration to counteract the pathophysiology of CMDs, there is an urgent need to better understand the molecular causes of these diseases to provide a basis for the development of effective therapeutic strategies. A promising approach to improve CMDs is cold-induced thermogenesis (CIT), which increases resting metabolic rate and energy expenditure and reduces body weight, adipocyte size, and plasma glucose concentrations [2]. In addition, CIT affects lipid metabolism by promoting the lipolytic clearance of triglyceride (TG)-rich VLDL by brown adipose tissue (BAT), leading to a reduction in circulating TG concentrations [3].

BAT is activated in response to cold stress and burns calories in the form of heat by uncoupling ATP synthesis from oxidative phosphorylation through the action of uncoupling protein 1 (UCP1). Cold exposure reduces body temperature, which triggers the release of norepinephrine in the sympathetic nervous system. This, in turn, activates the β3-adrenergic signaling pathway in thermogenic adipocytes. Cold stress also improves systemic cholesterol metabolism by inducing reverse cholesterol transport to the liver, HDL turnover, and the conversion of cholesterol to bile acids for elimination [4, 5]. In addition, ambient cold temperature leads to a reduction in hepatic glycogen and an increase in TG concentrations, gluconeogenic enzyme activities and glucose production, mitochondrial oxidation, and ketone body synthesis [6]. While CIT has been described as a potential treatment option against CMDs, pharmacotherapies in humans based on β3-adrenergic receptor agonists have been compromised by unfavorable side effects such as excessive increases in heart rate, blood pressure, and body temperature [7]. Therefore, effective strategies stimulating CIT without adverse side effects are needed.

Secreted factors released by BAT, known as batokines, coordinate various metabolic adaptations during CIT in an autocrine, paracrine or endocrine manner [8]. Investigating the BAT secretome, understanding the mechanisms by which this tissue communicates with others during CIT, and/or identifying factors that enhance the thermogenic capacity of BAT could be beneficial for the development of novel therapeutic approaches against CMDs. To date, numerous studies have examined the effects of CIT on individual cell types or tissues [6, 9]. Changes in various tissues at the transcriptomic level have also been described, as well as alterations in metabolic fluxes during acute cold stress in mice using metabolomics [10, 11]. To our knowledge, there are currently no reports on the effects of cold exposure on the BAT proteome in a CMD mouse model. We therefore fed LDL receptor-deficient (LdlrKO) mice a high-fat, high-sucrose, high-cholesterol diet (HFSCD) for 16 weeks [12]. We deemed this mouse model to be the most suitable for investigating the effects of cold exposure in metabolic tissues upon CMD, because it offers the advantage of mimicking the human disease while developing simultaneously all the complications associated with CMDs, such as white adipose tissue (WAT) inflammation, insulin resistance, and atherosclerosis. Many obesity models are resistant to atherosclerosis [13], whereas atherogenic apolipoprotein E KO mice fed a high-fat diet are less prone to develop diet-induced obesity, insulin resistance, and WAT inflammation than wild-type mice [14]. A predisposition to cardiometabolic dysfunction forms the backbone of metabolic dysfunction-associated liver disease [15], and metabolic liver disease is a significant risk factor for cardiovascular outcome [16]. In addition, the liver contributes to cold adaptation [17], rendering the liver an organ that fuels thermogenesis. However, most studies ignore the importance of the liver as a thermogenic organ and the potential beneficial effects of CIT on improving CMDs. By using shotgun proteomics on BAT and liver from HFSCD-fed, cold-exposed LdlrKO mice, we identified proteins differentially expressed in BAT and liver between room temperature (22 °C) and cold (5 °C). We discovered new pathways, transcription factors (TFs), miRNAs, and downstream target networks affected by cold exposure. Our findings also indicate that fibrinogen alpha chain (FGA) and fibronectin 1 (FN1) might mediate the crosstalk between BAT and liver during CIT. The robust associations between genetic variations in both genes and several cardiometabolic-related phenotypes and traits in humans suggest that they may be relevant for the development of CMDs in humans.

## Methods

### Animals and diets

Male LdlrKO mice (#002207, RRID: IMSR_JAX:002207; The Jackson Laboratory, Bar Harbor, ME) and C57BL/6J mice were housed in a clean and temperature-controlled environment (22 ± 1 °C; relative humidity 45–65%) with unlimited access to chow diet and water on a regular 12-hour light-dark cycle. At 6 weeks of age, the mice were fed a HFSCD (0.18% cholesterol, 58 kcal% fat (primarily lard), 28 kcal% carbohydrates (with 17.5 kcal% from sucrose); D09071704, Research Diets Inc, New Brunswick, NJ) for 16 weeks. Body weight was monitored weekly. At the end of the feeding period, one cohort of the LdlrKO mice were housed in automated metabolic cages (Phenomaster, TSE Systems, Bad Homburg, Germany) initially at 22 °C and then at 5 °C for 7 days. Energy expenditure, respiratory exchange ratio, food and water intake, and locomotor activity (using infrared sensor frames) were measured every 15 min. The mice were sacrificed after 12h of fasting. In addition, male LdlrKO and wild-type mice were fed chow diet and maintained at 5 °C or 22 °C, respectively. The mice were sacrificed at 22 °C or following 7 days at 5 °C in the fed state, and the organs were isolated.

All experiments were conducted in compliance with the European Directive 2010/63/EU and approved by the Austrian Federal Ministry of Education, Science and Research (Vienna, Austria; BMBWF-66.010/0138-V/3b/2019).

### Hepatic lipid concentrations

Lipids (triglycerides (TG), total cholesterol, free cholesterol, cholesteryl esters) were extracted and measured as previously described [18].

### RNA isolation and quantitative real-time PCR analysis

Total RNA was isolated using the TRISure™ reagent (Meridian, Memphis, TE) according to the manufacturer’s protocol. Two µg of total RNA were reverse transcribed to cDNA using the High-Capacity cDNA Reverse Transcription Kit (Thermo Fisher Scientific, Waltham, MA). Quantitative real-time PCR was performed on a CFX96 Real-Time PCR detection system (Bio-Rad Laboratories, Hercules, CA) using the GoTaq^®^ qPCR Master Mix (Promega, Madison, WI). Samples were normalized to the mRNA expression of cyclophilin A (*Ppia*) or β-actin (*Actb*) as reference genes. Expression profiles and associated statistical parameters were determined by the 2^−ΔΔCT^ method. Primer sequences are listed in Additional File 1, Supplementary Table S1.

### Western blotting

Liver and BAT samples were homogenized in RIPA buffer supplemented with protease/phosphatase inhibitor cocktail (1:1000; Merck, Darmstadt, Germany). Fifty or 25 µg of protein were separated by SDS-PAGE and transferred to PVDF membranes, which were incubated overnight with rabbit polyclonal antibodies against anti-FGA (1:1000; #20645-1-AP, RRID: AB_2878715, Proteintech, Planegg-Martinsried, Germany) and anti-fibronectin (FN1) (1:1000; #NBP1-91258; Novus Biologicals, Centennial, CO). Monoclonal mouse anti-β-actin (ACTB) (1:10,000; #A5316; Sigma Aldrich, St. Louis, MO) and rabbit anti-glyceraldehyde-3-phosphate dehydrogenase (GAPDH) (1:1000; #2118S; Cell Signaling, Danvers, MA) were used as loading controls. Secondary HRP-conjugated anti-rabbit (1:2500; #31460; ThermoFisher Scientific, Waltham, MA) and anti-mouse (1:1000; #P0260, Dako, Glostrup, Denmark) were visualized by enhanced chemiluminescence detection on a ChemiDoc™ MP imaging system (Bio-Rad Laboratories, Hercules, CA). FGA and FN1 expressions were quantified by densitometry (ImageJ^®^ Software, Version 1.51) and normalized to the expression of ACTB or GAPDH, respectively.

### FGA and FN1 ELISAs

FGA and FN1 concentrations in the plasma of chow diet-fed LdlrKO mice were determined by ELISA (FGA; A78074, antibodies.com, Stockholm, Sweden; FN1: ab210967, Abcam, Waltham, MA) according to the manufacturers’ protocols.

### Histological analyses

BAT, subcutaneous white adipose tissue (sWAT), and liver samples were fixed in 4% neutral-buffered formalin for 24 h. BAT and sWAT samples were then embedded in paraffin, whereas the liver samples were stored in 30% sucrose until cryosectioning. Section (7 μm) were cut and stained with hematoxylin and eosin (H&E). To visualize neutral lipids and nuclei, liver cryosections were stained with oil red O (ORO) and Mayer’s hematoxylin, respectively. Staining in BAT and sWAT sections was visualized using a digital slide scanner (Slideview VS200; Olympus, Tokyo, Japan) connected to an external LED source (Excelitas Technologies, X-Cite Xylis, Mississauga, Canada). Liver samples were imaged with an Olympus BX63 microscope equipped with a DP73 camera (Olympus).

### Untargeted proteomic analysis of BAT and liver

BAT and liver from LdlrKO mice housed at either 22 °C or 5 °C (*n* = 6/group) were homogenized in 8 M urea, Tris-HCl 0.1 M pH 8.5, supplemented with protease inhibitors at a ratio of 1:100 (# 5872 S; Cell Signaling, Danvers, MA), and processed as reported elsewhere [19]. For each BAT sample, the corresponding volume of 50 mM ammonium bicarbonate solution was added to 20 µg of total proteins (final pH 8.5, final volume 60 µl). The proteins were reduced by incubation with 3 µl of 100 mM DTT for 30 min at 55 °C. For protein alkylation, 6 µl of 150 mM iodoacetamide were added and incubated for 20 min in the dark at RT. Trypsin digestion (#T7575-1KT; Merck, Darmstadt, Germany; enzyme to protein ratio 1:20) was performed overnight at 37 °C and stopped by acidification with trifluoroacetic acid (final percentage 1%). The LC-MS/MS analyses were performed using an Ultimate 3000 nano-LC system connected to an Orbitrap Fusion Tribrid Mass spectrometer (Thermo Fisher Scientific, Waltham, MA) equipped with a nano-electrospray ion source. The pre-concentration of peptide mixtures, gradient elution, and MS spectra collection were previously described [20]. The data acquisition was operated in the data-dependent mode with a cycle time of 3 s between master scans. MS/MS spectra were collected in centroid mode. Higher collisional dissociation was performed with a collision energy set at 35 eV. The LC-MS/MS raw files were processed with an open-source, multiple peptide sequencing pipeline for label-free quantification (LFQ) based on OpenMS in the KNIME analytics platform as described [21]. Peptide indexing was performed against a Swiss-Prot verified FASTA protein sequence database for mice (uniprot-filtered-organism_Mus.musculus-(Mouse), *n* = 17,212 entries), downloaded at www.uniprot.org, which included a list of commonly contaminant proteins (*n* = 179, https://github.com/pwilmart/fasta_utilities/blob/master/Thermo_contams.fasta).

Data were processed and analyzed as previously described [22]. Volcano plots were generated using VolcaNoseR (https://huygens.science.uva.nl/VolcaNoseR2/) [23]. Ingenuity Pathway Analysis (IPA) (QIAGEN, Venlo, Netherlands) was performed based on the differentially expressed proteins (*p* < 0.05) [24].

### Analyses of secreted proteins and ligand-receptor pairs

To identify potentially secreted proteins among those significantly upregulated in BAT, we defined proteins as secreted based on the following criteria: (i) presence of a signal peptide as determined by SignalP-5.0 for eukaryotic amino acid sequences [25], (ii) annotation as non-classical secreted proteins with a score > 0.6 by SecretomeP-2.0 [26], (iii) annotation as secreted or extracellularly localized by LocTree3 [27], or (iv) annotation for subcellular localization as secreted or secretory vesicle in the Uniprot database [28]. Proteins annotated as localized in the mitochondrion, endoplasmic reticulum, or cytoplasm were considered non-secreted. Crosstalk between BAT and liver was identified by matching secreted and significantly induced ligands in BAT with corresponding receptors in the liver. Pairs were found by at least one positive hit in one of nine mouse ligand-receptor databases as summarized in [29], and ranked by the change of regularized product score [30] on mean protein abundances (log2 label-free quantitation) upon cold induction. Since log2 fold-changes to indicate induced ligands were based on normalized protein levels in the log2 space, we calculated the change of another score based on the sum of the mean of these normalized levels upon cold exposure.

### Networks of transcription factors, miRNAs, and downstream targets

The activation state of TFs and miRNAs was predicted by upstream regulator analysis from IPA based on dysregulated downstream targets (QIAGEN, Venlo, Netherlands) [24]. The TFs and miRNA with significant z-scores ≥ 2 or ≤ − 2 were considered for molecular network construction using Cytoscape software (Version 3.8.2, Seattle, WA) [31].

### Evaluation of human genetic support by genome-wide association studies (GWAS)

Human traits associated with genetic variations were investigated in individual GWAS studies using the gene symbols *FN1* and *FGA* in Open Target Genetics database (https://genetics-docs.opentargets.org/; accessed on Feb 26, 2024) [32, 33]. It includes published GWAS from the curated GWAS catalogue and newly derived GWAS from FinnGENN and UKBiobank. All SNP-trait associations from the relevant gene regions with *p*-value < 5 × 10^−8^, which is defined as genome-wide significant, for the index variant are retrieved. Furthermore, genetic variations in *FGA* or *FN1* and their associations with different phenotypes were also assessed with the Common Metabolic Diseases Knowledge Portal (CMDKP), which has the advantage of integrating multiple publicly available datasets and summarizing results from common and rare variants from the genes of interest into one score. Phenotypes were selected according to the proposed Human Genetic Evidence (HuGE) score (quantifying genetic support for the involvement of the specific gene with the trait) and its logarithmic correction (HuGE score ≥ 3; log_e_(HuGE score) ≥ 1.09, indicating at least moderate genetic support) [34]. For CMDKP, data were retrieved as follows:

Common Metabolic Diseases Knowledge Portal (cmdkp.org). *FGA* Gene page. 2024 Feb 15; https://hugeamp.org/gene.html?gene=FGA (RRID: SCR_020937).

Common Metabolic Diseases Knowledge Portal (cmdkp.org). *FN1* Gene page. 2024 Feb 15; https://hugeamp.org/gene.html?gene=FN1 (RRID: SCR_020937).

### Statistical analyses

Statistical analyses were performed using GraphPad Prism 9.3.1 software. Data are presented as mean ± SD, and differences between two groups were determined using the unpaired Student’s t-test. The following levels of statistical significance were used: *p < 0.05, **p ≤ 0.01, ***p ≤ 0.001.

## Results

### Increased metabolic activity, BAT function, and hepatic lipid content in cold-exposed LdlrKO mice

**Fig. 1:**
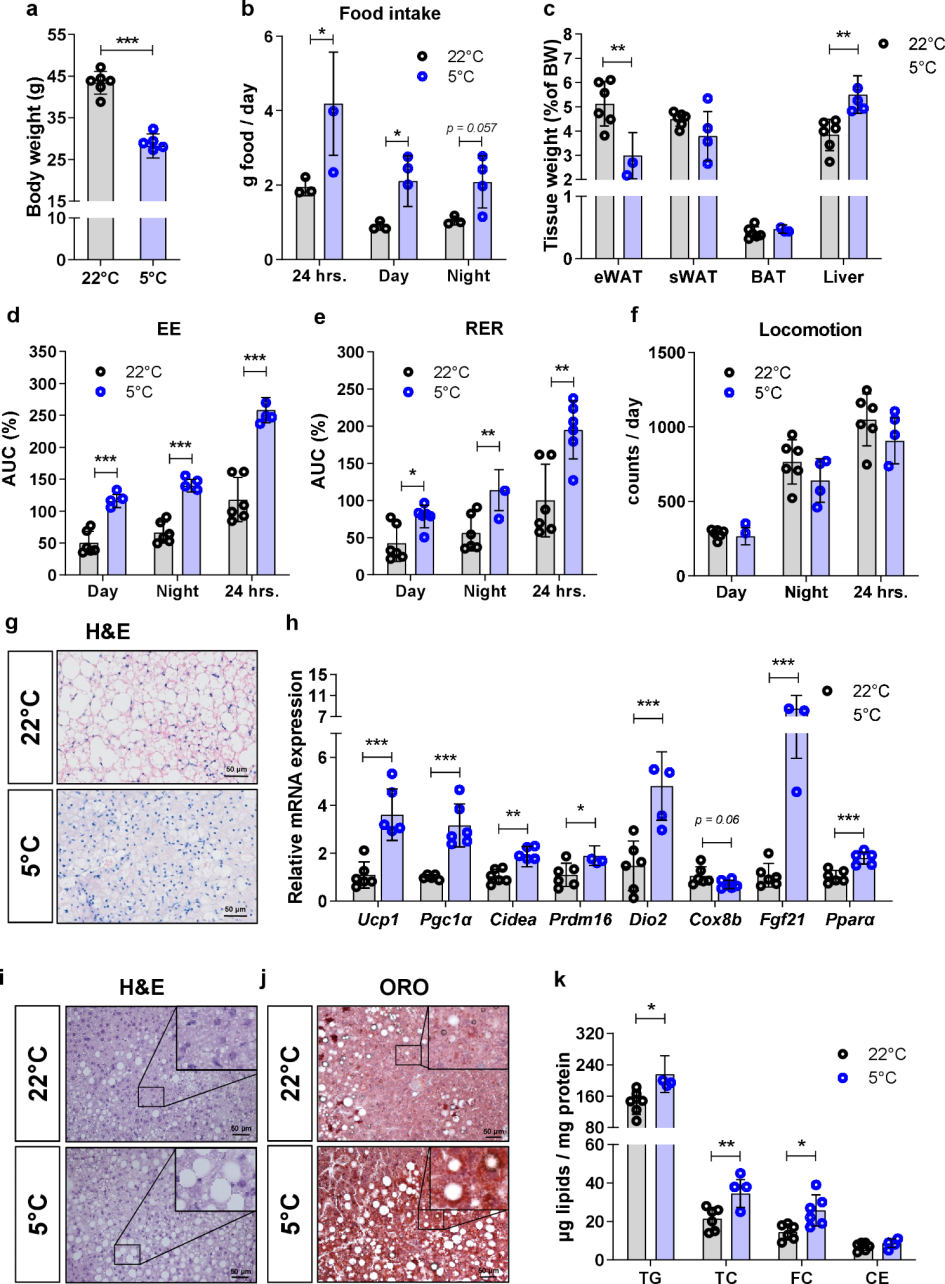
Increased metabolic activity, BAT function, and hepatic lipid deposition in LdlrKO mice after cold exposure. Male LdlrKO mice were fed HFSCD for 16 weeks and then housed at 22 °C or 5 °C for 7 days. **a** Body weight, **b** food intake, and **c** tissue weights before and after cold exposure. Area under the curve (AUC) calculated from **d** energy expenditure (EE) and **e** respiratory exchange ratio (RER) before and after cold exposure (the first 2 days and the last day were excluded from calculations, due to acclimation and fasting respectively). **f** Daily locomotor activity before and after cold exposure. **g** H&E staining of BAT sections (scale bar, 50 μm). **h** mRNA expression of browning genes in BAT relative to *Actb* expression. **i** H&E, **j** oil red O (ORO) staining of liver sections (scale bars, 50 μm), and **k** quantification of hepatic lipid concentrations. Data represent mean values (*n* = 6 except for (**b**) where food was placed directly into the cage for 5 mice (3 mice at 22 °C and 2 mice at 5 °C refused to eat constantly from the food container) ± SD; *p < 0.05, **p ≤ 0.01, ***p ≤ 0.001

We first performed in vivo energy metabolism analyses in our cardiometabolic mouse model using metabolic cages. After 7 days of cold exposure, LdlrKO mice showed a significant reduction in body weight by 37% (Fig. 1a) despite increased food intake (Fig. 1b). Cold-exposed mice showed a significant reduction in epididymal (e)WAT weight, sWAT and BAT remained unaltered, and liver mass was increased (Fig. 1c). Mice kept at 5 °C showed an increase in energy expenditure and respiratory exchange ratio (Fig. 1d, e; Additional File 1, Supplementary Fig. S1a, b) but comparable locomotion activity (Fig. 1f). H&E staining of BAT revealed considerably smaller lipid droplets (Fig. 1g), and the expression levels of several browning genes were significantly increased (Fig. 1h). We observed similar changes (reduced lipid droplet size, enhanced expression of browning markers) in sWAT from cold-exposed mice (Additional File 1, Supplementary Fig. S1c, d).

Activation of the thermogenic capacity in the WAT depots and thus lipolysis triggered by cold exposure leads to an augmented flux of fatty acids (FA) into BAT, but also into the liver, which in turn promotes hepatic lipid remodeling and TG accumulation [35]. Accordingly, the cold-exposed mice exhibited an increased hepatic gene expression of the FA transporter *Cd36* (Additional File 1, Supplementary Fig. S1e) and hepatic steatosis, as shown by neutral lipid staining (Fig. 1i, j) and quantification of intracellular TG content (Fig. 1k), which could explain the increased liver weight as a consequence of metabolic adaptation to increased food intake. Altogether, these data showed substantial metabolic effects in BAT and liver of HFSCD-fed LdlrKO mice in response to cold exposure.

### Cold exposure alters the proteome of BAT and liver from LdlrKO mice

**Fig. 2:**
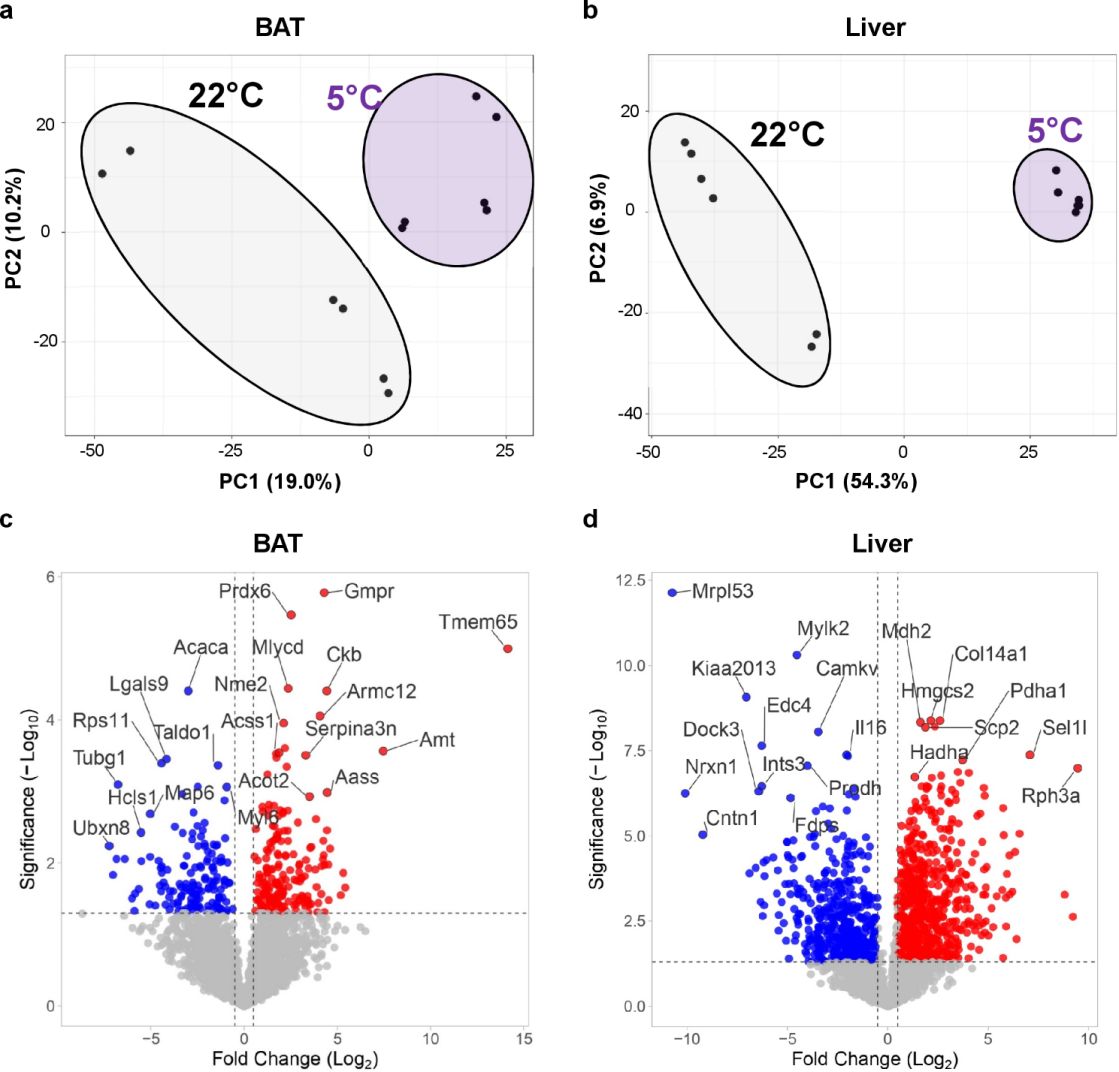
Altered proteomes in BAT and liver from LdlrKO mice after cold exposure. Male LdlrKO mice were fed HFSCD for 16 weeks and then housed at 22 °C or 5 °C for 7 days. Principal component analysis plots of **a** BAT and **b** liver (*n* = 6). Volcano plots showing upregulated (red) and downregulated proteins (blue) by cold exposure in **c** BAT and **d** liver

To identify changes in the proteome signature of BAT and liver of HFSCD-fed LdlrKO mice by cold exposure, we performed shotgun proteomics. Principal component analysis revealed a distinct clustering of samples collected at 22 °C and 5 °C (Fig. 2a, b). In BAT, among 2979 proteins quantified, 330 were significantly differentially expressed (*p* < 0.05) in mice maintained at 5 °C, with 184 upregulated (Log_2_FC > 0.5) and 146 downregulated (Log_2_FC < − 0.5) (Fig. 2c; Additional File 2). Among the most dysregulated proteins in BAT, ACACA, TALDO1, LGALS9, MAP6, HCLS1, UBXN8, RPS11, TUBG1, and MYL6 were downregulated, whereas PRDX6, GMPR, TMEM65, MLYCD, CKB, ARMC12, ACSS1, NME2, SERPINA3N, AMT, ACOT2, and AASS were upregulated as highlighted in the volcano plot (Fig. 2c). Consistent with the phenotype, we also detected an upregulation of UCP1 and of several proteins involved in FA metabolism and β-oxidation (ACADSB, HSD17B4, HSD17B8, and ACOX1) as well as the downregulation of the lipid synthesis-related enzyme FASN in BAT (Additional File 1, Supplementary Fig. S2a, b). Of the 2329 quantified proteins in the liver, 1063 were significantly differentially expressed (*p* < 0.05), of which 587 were upregulated (Log_2_FC > 0.5) and 442 were downregulated (Log_2_FC < − 0.5) (Fig. 2d; Additional File 3). The downregulated proteins in the liver included MRPL53, MYLK2, KIAA2013, EDC4, IL16, CAMKV, DOCK3, NRXN1, INTS3, PRODH, FDPS, and CNTN1, whereas the top upregulated proteins were MDH2, HMGCS2, COL14A1, PDHA1, SCP2, HADHA, SEL1L, and RPH3A (Fig. 2d). In addition, we found increased protein abundance of enzymes involved in lipid uptake, transport, synthesis, and storage (CD36, SLC27A2, FABP5, ACSL5, ACSL1, PCK1, and PLIN2) (Additional File 1, Supplementary Fig. S2c), consistent with increased hepatic TG levels. Collectively, these results indicate that cold exposure induces profound changes in the proteome and in the regulation of lipid metabolism in BAT and liver of our cardiometabolic mouse model.

### Cold exposure affects molecular processes and functions in BAT and liver of LdlrKO mice

**Fig. 3:**
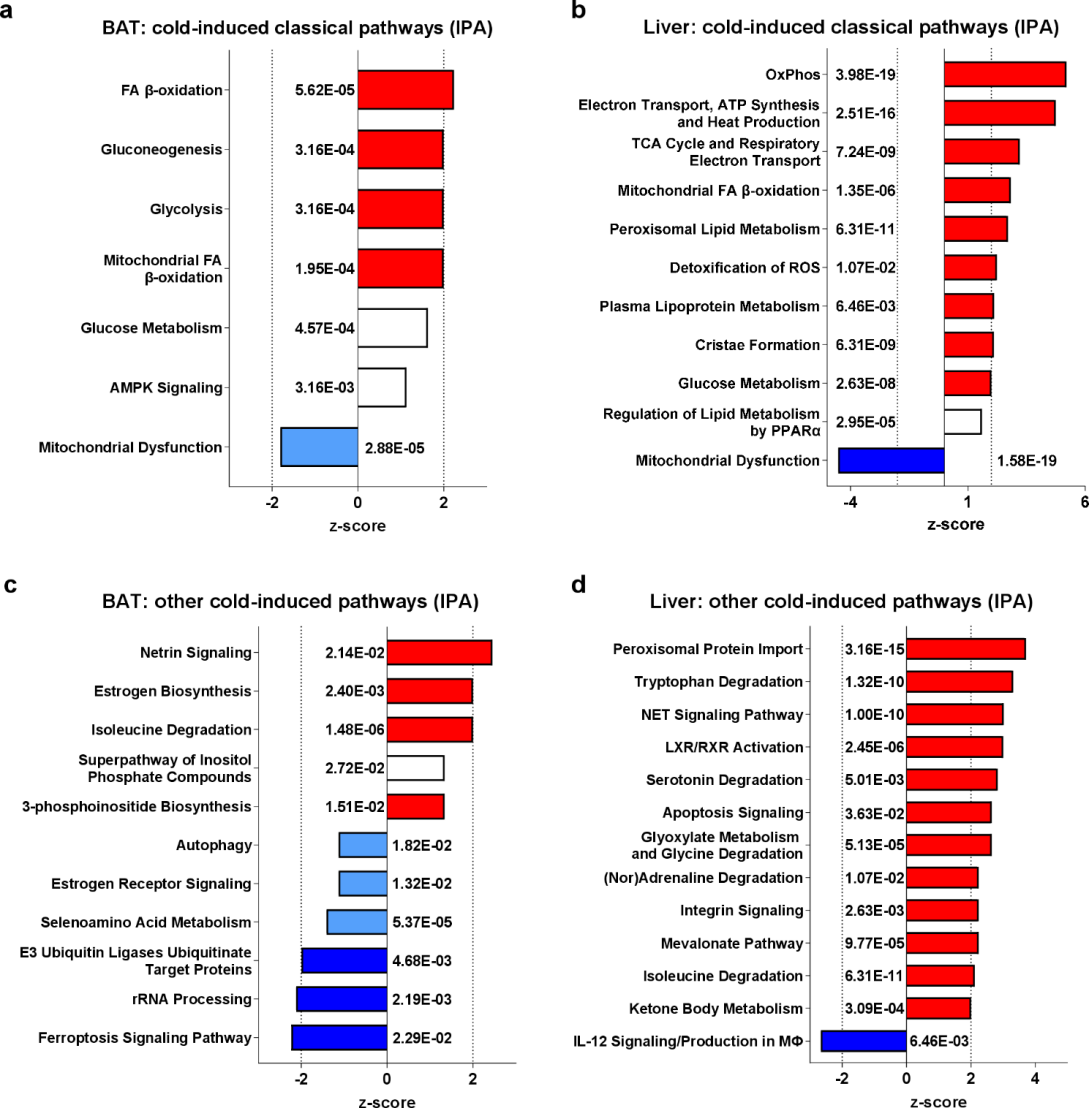
Cold-induced classical and non-classical pathways in BAT and liver of LdlrKO mice. Male LdlrKO mice were fed HFSCD for 16 weeks and then housed at 22 °C or 5 °C for 7 days. Ingenuity Pathway Analysis (IPA) of significant proteins showing cold-induced classical pathways in **a** BAT and **b** liver and other pathways in **c** BAT and **d** liver. Downregulated pathways in cold-exposed mice are shown in blue (z-score > − 2 in light blue; z-score ≤ − 2 in dark blue), upregulated pathways are shown in red (z-score < 2 in light red; z-score ≥ 2 in dark red). *p*-values are indicated next to each bar

Next, we performed pathway analysis in IPA to identify the molecular processes and functions associated with the changes in the proteomes. Dysregulation of several classical cold-induced pathways in BAT and liver further confirmed the validity of our model (Fig. 3a, b). These included downregulation of mitochondrial dysfunction (i.e. upregulation of mitochondrial activity) and upregulation of FA β-oxidation in both tissues (Fig. 3a, b; Additional Files 2 and 3) as well as upregulation of gluconeogenesis in BAT and oxidative phosphorylation, electron transport, ATP synthesis and heat production, TCA cycle, peroxisomal lipid metabolism, detoxification of ROS, and plasma lipoprotein metabolism in the liver (Fig. 3b; Additional File 3).

Besides these classical pathways affected by cold exposure, we identified netrin signaling, estrogen biosynthesis, and isoleucine degradation as upregulated pathways in BAT (Fig. 3c; Additional File 2). Downregulated pathways were associated with ubiquitination, rRNA processing, and ferroptosis signaling (Fig. 3c; Additional File 2). In the liver, we also detected peroxisomal protein import, NET signaling, LXR/RXR activation, apoptosis, integrin signaling, and metabolism/catabolism of amino acids, serotonin, (nor)adrenaline, mevalonate, and ketone bodies as additional upregulated pathways upon cold exposure. In contrast, IL-12 signaling/production in macrophages was downregulated (Fig. 3d; Additional File 3). Taken together, these data demonstrated that the cold-mediated changes in the BAT and liver proteome might impact metabolic processes and molecular functions that are highly relevant to the pathogenesis of CMDs.

### **Dysregulation of TFs and miRNAs in BAT and liver of cold-exposed LdlrKO mice**

**Fig. 4:**
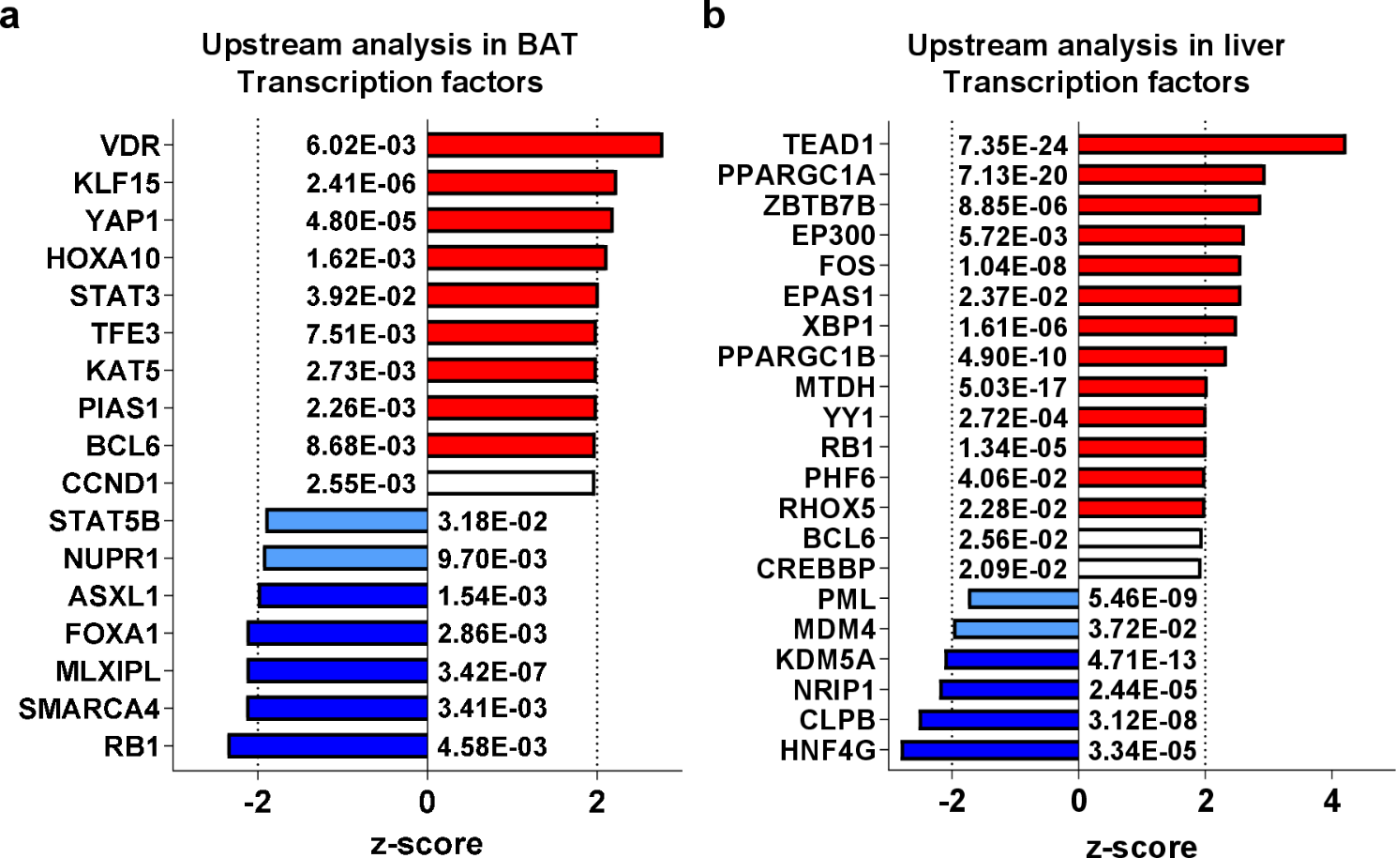
Dysregulation of transcription factors in BAT and liver of LdlrKO mice after cold exposure. Male LdlrKO mice were fed HFSCD for 16 weeks and then housed at 22 °C or 5 °C for 7 days. Ingenuity Pathway Analysis (IPA) of dysregulated transcription factors in **a** BAT and **b** liver. Transcription factors predicted to be inhibited in cold-exposed mice are shown in blue (z-score > − 2 in light blue; z-score ≤ − 2 in dark blue), transcription factors predicted to be activated are shown in red (z-score < 2 in light red; z-score ≥ 2 in dark red). *p*-values are indicated next to each bar

Using detailed upstream analyses in IPA, we identified numerous TFs and miRNAs with dysregulated activation states. Among the top activated TFs in BAT, we found VDR, KLF15, YAP1, HOXA10, STAT3, TFE3, KAT5, and PIAS1, whereas RB1, SMARCA4, MLXIPL, FOXA1, and ASXL1 were inhibited in mice housed at 5 °C (Fig. 4a; Additional File 2). In the liver, TEAD1, PPARGC1A, ZBTB7B, EP300, FOS, EPAS1, XBP1, PPARGC1B, MTDH, YY1, RB1, PHF6 and RHOX5 were predicted to be activated, whereas HNF4G, CLPB, NRIP1, and KDM5A were predicted to be inhibited (Fig. 4b; Additional File 3). miRNA predictions in BAT suggested the inhibition of miR-9-5p and miR-16-5p (Table 1; Additional File 2). In the liver, miR-9-5p and miR-1 were predicted to be the most strongly inhibited miRNAs in cold-exposed mice (Table 1; Additional File 3).

**Fig. 5:**
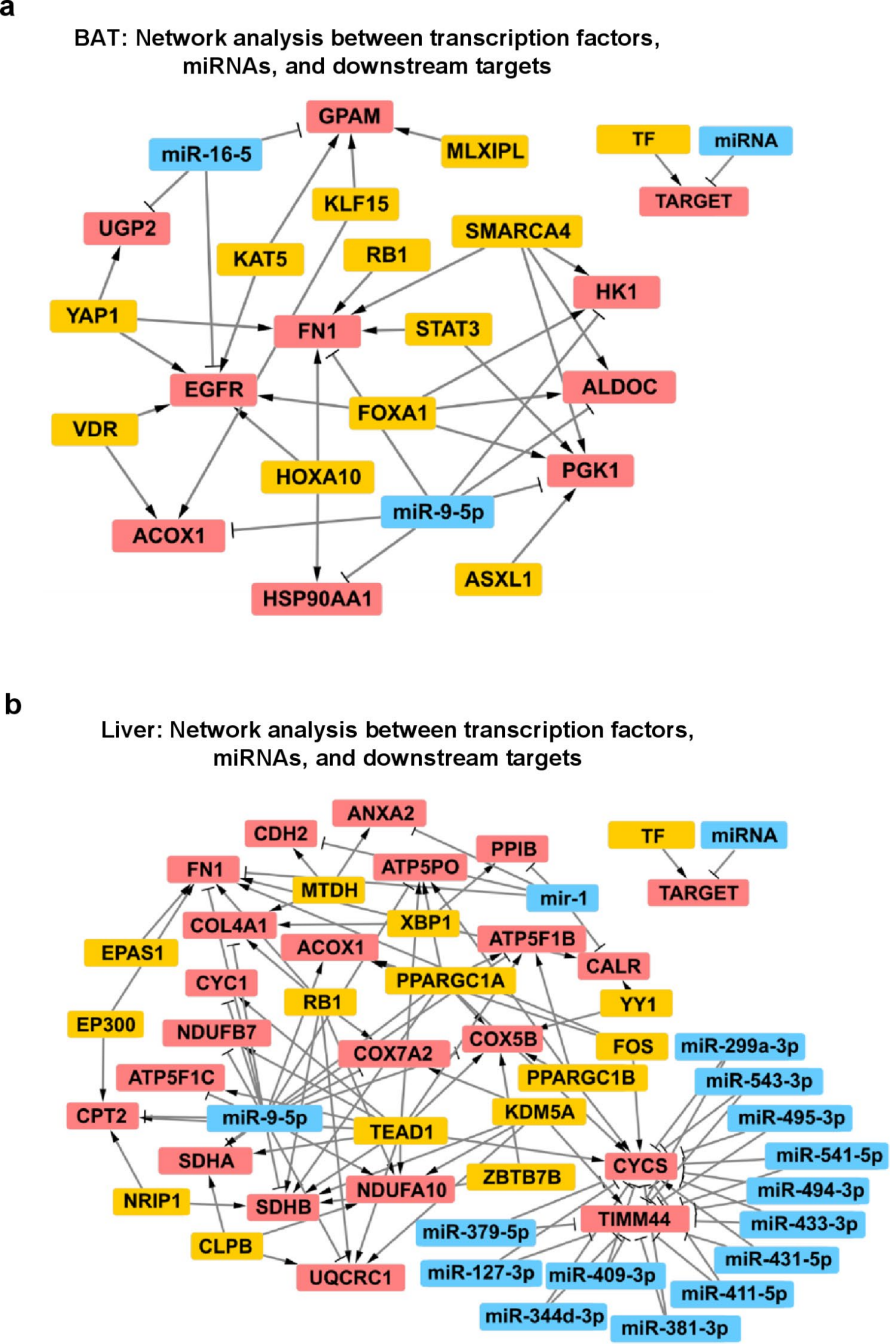
Network analyses of transcription factors, miRNAs, and downstream targets in BAT and liver of LdlrKO mice. Male LdlrKO mice were fed HFSCD for 16 weeks and then housed at 22 °C or 5 °C for 7 days. Transcription factors (TF), miRNAs, and their downstream targets determined by upstream analysis in IPA were used to build molecular networks for **a** BAT and **b** liver. The networks were created using Cytoscape software

To investigate potential connections between the identified TFs and miRNAs in BAT and liver, we created molecular networks based on their common downstream targets. In BAT, we detected SMARCA4 and FOXA1 as the TFs with the highest number of potential interactions with downstream targets (Fig. 5a; Additional File 4). miR-16-5p might inhibit UGP2, GPAM, and EGFR, and miR-9-5p might target FN1, HK1, ALDOC, PGK1, and HSP90AA1 (Fig. 5a; Additional File 4). Upon closer analysis, we identified EGFR, FN1, GPAM, and PGK1 as downstream targets of multiple TFs and miRNAs and as connectors of several sub-networks (Fig. 5a; Additional File 4). In the liver, we found TEAD1, RB1, MTDH, XBP1, FOS, PPARGC1A, PPARGC1B, and KDM5A to be regulators of the downstream targets identified by proteomics (Fig. 5b; Additional File 4). Moreover, 13 of the 15 miRNAs predicted to be inhibited after cold exposure were linked to TIMM44 and CYCS, which were central in the network since they connected the largest number of regulators. Thus, these findings uncovered tissue-specific TFs, miRNAs, and their interactions with downstream targets that are activated or inhibited by cold. Future studies are needed to validate this in silico model and experimentally determine the activation states of TFs and miRNAs.

**Table 1:** List of dysregulated miRNAs in BAT and liver of HFSCD-fed LdlrKO mice after cold exposure

Name	Activation z-score	Predicted activation state	*p*-value
BAT			
miR-9-5p	− 2.40	Inhibited	2.35E−04
miR-16-5p	−2.19	Inhibited	7.06E−05
Liver			
miR-9-5p	− 4.01	Inhibited	5.41E−13
mir-1	− 2.93	Inhibited	2.91E−02
miR-127-3p	− 2.00	Inhibited	1.54E−03
miR-299a-3p	− 2.00	Inhibited	8.56E−04
miR-344d-3p	− 2.00	Inhibited	8.56E−04
miR-379-5p	− 2.00	Inhibited	2.00E−03
miR-381-3p	− 2.00	Inhibited	3.18E−03
miR-409-3p	− 2.00	Inhibited	6.09E−04
miR-411-5p	− 2.00	Inhibited	8.56E−04
miR-431-5p	− 2.00	Inhibited	8.56E−04
miR-433-3p	− 2.00	Inhibited	2.00E−03
cmiR-494-3p	− 2.00	Inhibited	4.76E−03
miR-495-3p	− 2.00	Inhibited	2.00E−03
miR-541-5p	− 2.00	Inhibited	4.18E−04
miR-543-3p	− 2.00	Inhibited	1.16E−03

### FGA and FN1 are potential candidates for crosstalk between BAT and liver after cold exposure

Next, we searched for ligand-receptor pairs that could mediate crosstalk between BAT and liver during cold exposure. To identify potential ligands for receptors in the liver, we preselected all upregulated proteins in the BAT known to be secreted and their specific receptors in the liver in accordance with at least one of nine ligand-receptor databases (Additional File 5). Ligand-receptor pairing yielded several hits, of which we identified CLU, FGA, FN1, HPX, and complement (C3) as upregulated ligands with matching receptors in the liver, which were also increased upon cold exposure (Table 2).

**Table 2:** Ligand-receptor interactions between BAT and liver of LdlrKO mice during cold exposure

BAT ligand	Fold change	Liver receptor	ΔLR score	ΔSum score	No. of LRdb
CLU	2.81	LRP2	0.014	1.07	1
**FGA**	**2.74**	**PLAT**	**0.035**	**4.58**	**1**
**FGA**	**2.74**	**ITGB2**	**0.029**	**3.85**	**3**
**FGA**	**2.74**	**ITGB1**	**0.021**	**2.67**	**4**
FGA	2.74	ITGAV	0.016	1.52	2
FGA	2.74	CDH5	− 0.005	− 2.51	1
**FN1**	**2.01**	**ITGB2**	**0.028**	**3.40**	**1**
**FN1**	**2.01**	**ITGB1**	**0.020**	**2.22**	**4**
FN1	2.01	DPP4	0.017	1.69	4
FN1	2.01	MAG	0.012	1.08	6
FN1	2.01	ITGAV	0.015	1.07	7
FN1	2.01	FLT4	0.000	− 1.83	1
FN1	2.01	ITGA6	− 0.004	− 2.61	5
HPX	1.77	LRP1	0.011	0.22	2
C3	1.65	ITGB2	0.027	3.12	1
C3	1.65	LRP1	0.011	0.12	5
C3	1.65	CFB	0.007	− 0.51	6

Fold change: fold change of the ligand in BAT upon cold induction; No. of LRdb: evidence for a ligand-receptor interaction in this number out of 9 ligand-receptor databases; ΔLR score: change in regularized product score upon cold induction; Sum score: change in the sum of normalized log2 levels for ligand in BAT and receptor in liver upon cold induction; BAT ligands with > 2-fold change and ΔLR score > 0.02 (Sum score > 2) are indicated in bold

**Fig. 6:**
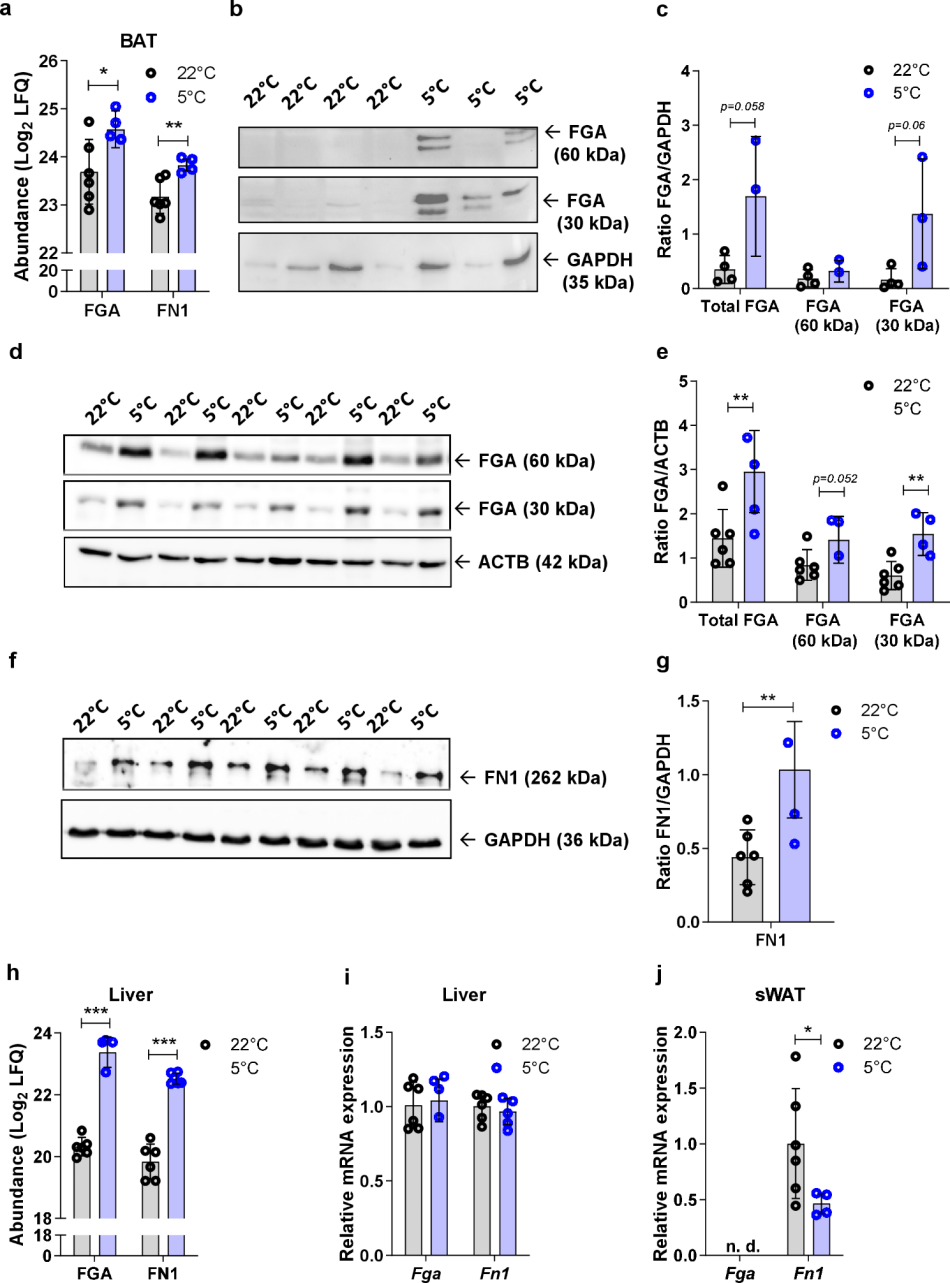
FGA and FN1 are increased in the liver of cold-exposed LdlrKO mice. Male LdlrKO mice were fed HFSCD for 16 weeks and then housed at 22 °C or 5 °C for 7 days. **a** Relative log2 abundance of FGA and FN1 from the BAT proteomics data. Data represent mean values (*n* = 6) ± SD; *p < 0.05, **p ≤ 0.01. **b** Western blotting experiment of FGA protein expression in BAT with β-actin (ACTB) as loading control and the **c** densitometric quantification of FGA relative to ACTB. Data represent mean values (*n* = 4) ± SD; *p < 0.05, **p ≤ 0.01. **d** Hepatic protein expression of FGA and **f** FN1 with β-actin (ACTB) and glyceraldehyde-3-phosphate dehydrogenase (GAPDH) as loading controls, respectively. Densitometric quantification of **e** FGA and **g** FN1 relative to the expression of the respective control. **h** Log2 protein abundance of FGA and FN1 from the liver proteomics datasets. **i** mRNA expression of *Fga* and *Fn1* in liver and **j** sWAT relative to *Ppia* expression. Data represent mean values (*n* = 5–6) ± SD; *p < 0.05, **p ≤ 0.01, ***p ≤ 0.001

For further validation analyses, we selected those ligands with a fold change of > 2 in BAT upon cold induction and a matched receptor in the liver with a change in regularized product score (ΔLR score) > 0.02 (Δsum score > 2) upon cold induction, which reflects the combined abundance of the receptors and their ligands. This analysis yielded 5 distinct pairs (FGA-PLAT, FGA-ITGB1, FGA-ITGB2, FN1-ITGB1, FN1-ITGB2), with FGA and FN1 emerging as potential candidates that may signal from the BAT to the liver in response to cold exposure (Table 2; Additional File 5). We reanalyzed the data and confirmed the increased abundance of the proteins in the BAT proteomics datasets (Fig. 6a) and by Western blotting of FGA (Fig. 6b, c). Western blotting also confirmed the increased expression of FGA and FN1 in the liver as the potential target tissue after cold exposure (Fig. 6d–g), consistent with the proteome analysis of the same samples (Fig. 6h). Moreover, the comparable hepatic gene expression of *Fga* and *Fn1* at 22 °C and 5 °C indicated that the observed differences in FGA and FN1 protein expression in the liver were independent of autonomous tissue effects resulting from cold exposure (Fig. 6i). Given that sWAT also undergoes browning in response to cold exposure, we assessed the transcriptional levels of *Fga* and *Fn1* at 5 °C and 22 °C in WAT. *Fga* was not expressed, whereas *Fn1* was even downregulated in sWAT upon cold (Fig. 6j). In skeletal muscle as another tissue that might be activated by cold, *Fga* and *Fn1* mRNA were low expressed (C_T_ values > 30; data not shown). Protein abundance of FGA and FN1 in skeletal muscle and kidney, two of the tissues with highest release of FGA and FN1 in exercised mice [36], was low under both conditions and unaffected by cold exposure (data not shown). We next investigated whether our findings were independent of HFSCD and/or LDLR deficiency and may also be observed in wild-type mice. We therefore determined the protein abundance of FGA in the liver of LdlrKO and wild-type mice fed chow diet as well as in control mice that were fed HFSCD for 16 weeks and then kept at 22 °C or 5 °C for 7 days. Of note, the increased abundance of FGA (30 kDa) was similar to that of the cardiometabolic mice, whereas FGA (60 kDa) was comparable between 22 °C and 5 °C (Additional File 1, Supplementary Fig. S3a-f). Circulating FGA levels of chow-diet fed LdlrKO mice after 7 days at 22 °C or 5 °C were only slightly but significantly increased by cold exposure (Additional File 1, Supplementary Fig. S3g), whereas FN1 plasma levels were comparable between both conditions (Supplementary Fig. S3h). These findings indicate that a cardiometabolic trigger is necessary for their increased release in the circulation.

**Fig. 7:**
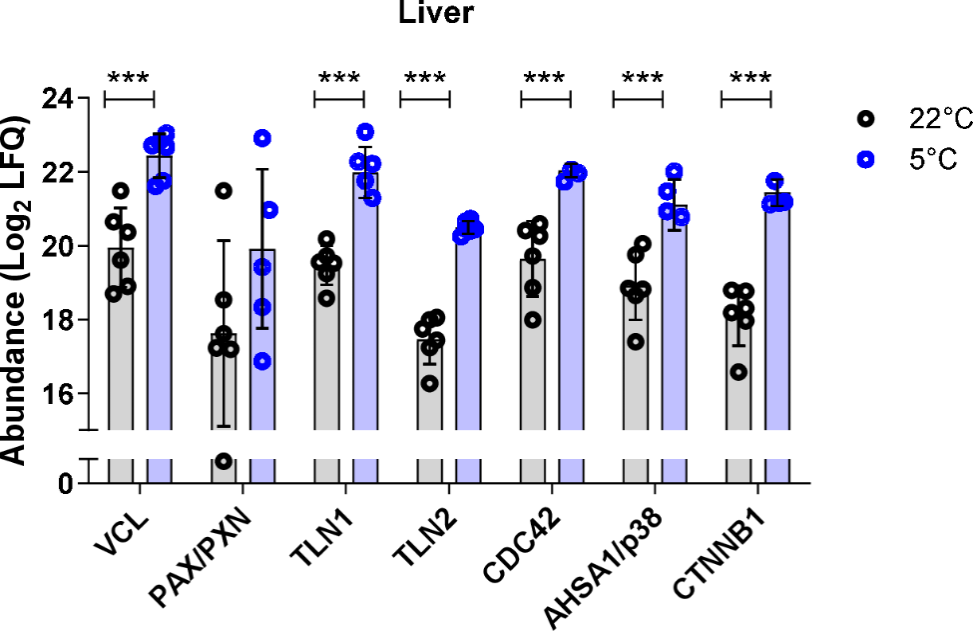
Increased protein abundance of ITGB1 downstream targets in the liver of cold-exposed LdlrKO mice. Male LdlrKO mice were fed HFSCD for 16 weeks and then housed at 22 °C or 5 °C for 7 days. Potential activation of ITGB1 as a common liver receptor for FGA and FN1 shown by log2 protein abundance of ITGB1 downstream targets (vinculin (VCL), paxillin (PAX/PXN), talin (TLN1 &TLN2), cell division cycle 42 (CDC42), activator of heat shock protein ATPase 1 (AHSA1/p38), and catenin beta 1 (CTNNB1)) from the liver proteomics datasets. Data represent mean values (*n* = 6) ± SD; ***p ≤ 0.001

Given that ITGB1 emerged as one of the common hepatic receptors for both FGA and FN1, we selected ITGB1 as a receptor for further investigation into potential downstream target activation. Several proteins previously described in the signaling cascade of ITGB1, such as vinculin (VCL), paxillin (PAX/PXN), talin (TLN1 &TLN2), cell division cycle 42 (CDC42), activator of heat shock protein ATPase 1 (AHSA1/p38), and catenin beta 1 (CTNNB1), were increased in the liver of cold-exposed LdlrKO mice (Fig. 7). Collectively, these findings support the link between cold exposure, activated BAT, and increased hepatic FGA and FN1 protein expression.

### Variations in *FGA* and *FN1* are linked to cardiometabolic-related phenotypes and traits in humans

**Fig. 8:**
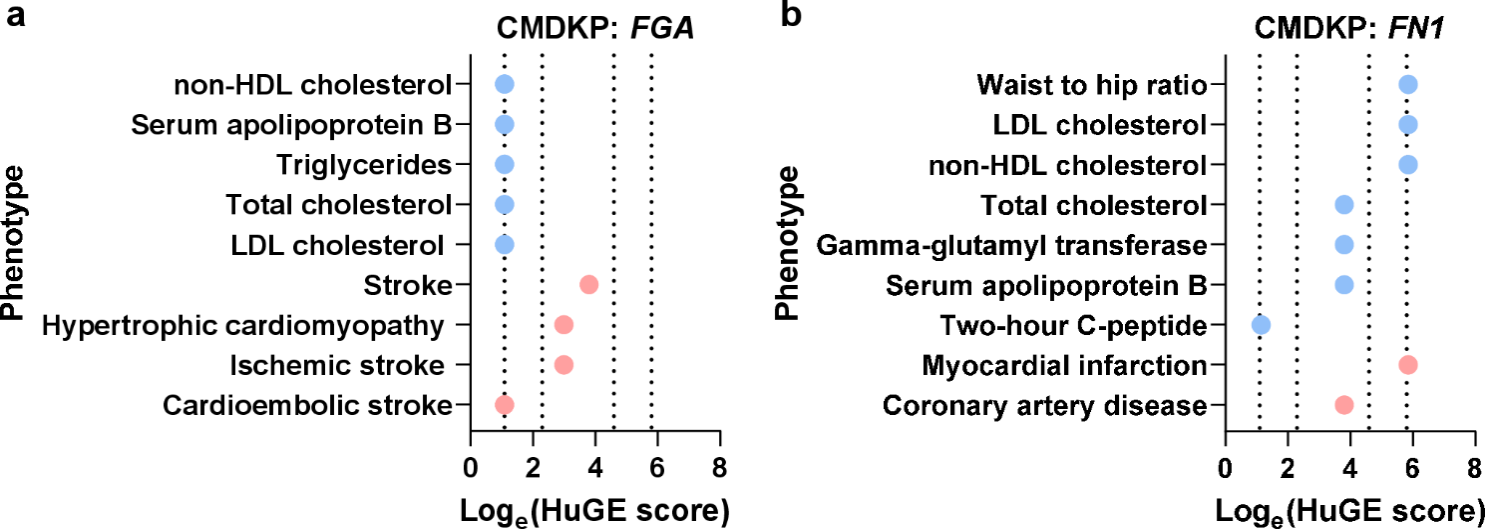
Genetic variations in FGA and FN1 are associated with cardiometabolic phenotypes in humans. Phenotypes related to genetic variations in **a***FGA* and **b***FN1* according to the Common Metabolic Diseases Knowledge Portal (CMDKP). Cardiovascular- and metabolic-related phenotypes are shown in red and blue, respectively. Support for genetic evidence is defined as: Moderate: Log_e_(HuGE score) ≥ 1.09; Strong: Log_e_(HuGE score) ≥ 2.30; Extreme: Log_e_(HuGE score) ≥ 4.60; Compelling: Log_e_(HuGE score) ≥ 5.86. The vertical dotted lines indicate the thresholds between evidence categories

Finally, we investigated the potential relevance of FGA and FN1 in CMDs in humans by analyzing whether genetic variations in their genes are associated with cardiometabolic-related phenotypes and traits. Exploring the CMDKP revealed that variations in both *FGA* and *FN1* were strongly associated with several cardiometabolic-related phenotypes in humans. Genetic alterations in *FGA* were linked to different types of strokes, cardiomyopathy, and the circulating concentrations of cholesterol and TG (Fig. 8a; Additional File 6). Variations in *FN1* were related to coronary artery disease, myocardial infarction, circulating concentrations of cholesterol, apolipoprotein B, C-peptide, and waist-to-hip ratio with even “very strong” or “compelling” evidence of genetic support (Fig. 8b; Additional File 6). In addition, these metadata from CMDKP were supported by data from individual studies available in the Open Targets Databases. All significantly associated human traits with variants within both genes are listed in Additional File 1, Supplementary Fig. 4a, b; Additional File 6. The most significant traits were fibrinogen levels, venous thromboembolism, LDL and TG levels, and stroke for *FGA*. For *FN1*, significant traits included serum and metabolite levels and parameters of blood cell count, but also cardiometabolic traits with very high genetic support, like blood pressure, coronary artery disease, and waist-hip-ratio. In summary, these observations indicate that FGA and FN1 might also be relevant to CMDs in humans.

## Discussion

BAT activation has gained considerable attention since its discovery in adult humans over a decade ago due to its ability to dissipate energy and counteract CMDs. The identification of the molecular mediators, the regulatory basis, and the modulation of the signaling network of BAT activation represents a promising therapeutic potential. Consistent with previous research on various animal models and humans [37], we found that cold exposure had profound systemic and local metabolic effects on the BAT and liver of HFSCD-fed LdlrKO mice. For example, ACACA, which catalyzes the rate-limiting step of *de novo* lipogenesis, was one of the most downregulated proteins in BAT. It is remarkable that thermogenic adipocytes express high levels of enzymes for both lipolysis and lipogenesis simultaneously [3]. However, cold-induced activation of the adrenergic signaling pathway leads to increased lipolysis, β-oxidation, and BAT hyperplasia and hypertrophy, which represent an adaptive response of rodents to survive in cold environments [3]. Despite increased food intake, body and eWAT weights were decreased but BAT weight was comparable between LdlrKO mice kept at 22 °C or 5 °C. This finding might be due to the chronic high-fat diet feeding that led to BAT whitening characterized by enlarged lipid droplets [38] under both conditions. The increased liver weight is a consequence of metabolic adaptation to increased food intake [39]. We can only speculate that the remaining weight change may be due to other adipose tissue depots (including perirenal ATs, mesenteric white AT, mediastinal BAT) and various skeletal muscles that we did not quantify when the mice were sacrificed.

Shotgun proteomics of BAT and liver revealed significant alterations in the proteome of both tissues in response to cold. As expected, we observed an increase in the abundance of several mitochondrial proteins in both tissues. TMEM65 was the most upregulated protein in BAT, and previous studies have shown that this protein has beneficial effects on oxidative stress and mitochondrial unfolded protein response and plays a crucial role in regulating mitochondrial dynamics [40, 41]. These findings suggest that TMEM65 might be a promising target involved in BAT activation and CIT. Canonical pathway analysis identified several metabolic processes and molecular functions in BAT and liver that were regulated by cold exposure. The netrin signaling pathway, which has not been previously described in the context of CIT, was the most upregulated pathway in BAT. The netrin protein family is a group of secreted ligands known to be involved in axon guidance in the nervous system, in the control of sympathetic innervation in arteries, and in blood flow regulation [42]. Activation of the netrin signaling pathway could also lead to increased sympathetic innervation and the release of norepinephrine in BAT upon cold exposure. Alternatively, this signaling pathway could mediate a sympathetic effect in the BAT vasculature, leading to vasodilation and increased release of catecholamines, ultimately resulting in CIT. However, it is unclear whether one or both scenarios apply and the exact effects of the netrin signaling pathway on CIT need to be clarified in the future. Among the downregulated pathways in BAT, the ferroptosis signaling pathway was the most prominent. Ferroptosis is an iron-dependent mechanism of cell death that is triggered by the accumulation of lipid hydroperoxides, dysregulation of iron-redox homeostasis, and impaired mitochondrial respiration. As a novel form of cell death, ferroptosis is increasingly recognized as playing a central role in the development of insulin resistance and pancreatic impairment [43, 44], myocardial dysfunction [45], and metabolic-associated fatty liver disease [46]. Consistent with a suggested link between ferroptosis and impaired thermogenesis [47, 48], cold exposure led to decreased activation of the ferroptosis signaling pathway in BAT.

In the livers of mice maintained at 5 °C, we found increased peroxisomal protein import, decreased mitochondrial dysfunction, and reduced IL-12 signaling and production in macrophages. Peroxisomes play an important role in the β-oxidation of very long-chain FA. Cold exposure reduces insulin and increases norepinephrine concentrations in the circulation, triggering neutral lipolysis in WAT. This results in FA secretion into the plasma and their uptake by the liver, which activates PPARα as well as mitochondrial and peroxisomal β-oxidation [49]. IL-12 is a pro-inflammatory cytokine secreted by classically activated macrophages and Kupffer cells [50], which has been associated with the development of hepatosteatosis [51]. The downregulation of only IL-12 signaling and production in the livers of cold-exposed HFSCD-fed LdlrKO mice, without any effect on other interleukins or inflammatory pathways, suggests the need for further investigation of the role of the IL-12 pathway in CMD. While wild type mice exposed to 4 C for 3 h per day for 3 weeks showed activation of ER stress and an increase in IL-1β and IL-6 [52], this was not observed in our experimental setting. Differences in genotype, diet, and/or duration of cold exposure may have contributed to this discrepancy. Nevertheless, our data indicate that 7 days of cold exposure can improve metabolic features in the liver of CMD mice. Future studies are needed to investigate the signals responsible for upregulating lipid transporters that may have caused steatosis in our model and whether a slight increase in hepatic steatosis is beneficial for improving CMD. We speculate that the observed beneficial effects may be linked to increased PPARα activation, which is known to reduce the production of pro-inflammatory cytokines [53].

Given the important role of TFs and miRNAs in transcriptional and post-transcriptional gene regulation, it was not surprising that we found numerous TFs and miRNAs with dysregulated activation states in BAT and liver upon cold exposure. Constructing molecular interaction networks from the data revealed novel regulatory relationships with higher biological relevance than investigating single motifs of miRNAs, TFs, and downstream targets. In both tissues, we identified several nodes with a striking number of connections, also referred to as hubs [54]. FN1, EGFR, PGK1, and GPAM were the targets with the highest number of regulatory connections in BAT. In the liver, CYCS and TIMM44 were the central targets of well-defined hubs and exhibited the strongest explanatory power and biological relevance within our network. Since we analyzed interactions between TFs or miRNAs with their downstream targets as feedforward loops [55], additional studies are necessary to classify the feedforward loops as either coherent (same direction, i.e. activation or repression) or incoherent (opposing directions). About 60% of miRNAs are encoded in polycistronic clusters, meaning they are co-transcribed with their cluster partners and act as cooperative units on the same gene [56]. We were unable to detect any polycistrons in the BAT due to the low number of miRNAs. However, we identified two polycistrons that corresponded to the hubs in the liver described above, emphasizing the regulatory capacity of these motifs, which should be investigated in more detail in the future.

Given that the primary responsive tissue to cold induction is BAT, we conducted further bioinformatic analyses to identify pairs between ligands secreted from BAT and their corresponding receptors in the liver. The most promising candidate molecules that may mediate crosstalk between BAT and liver during CIT were the secreted glycoproteins FGA and FN1. We detected an increase in hepatic FGA and FN1 protein expression following cold exposure but no transcriptional regulation in the liver, skeletal muscle, or sWAT. Thus, despite the liver exhibiting the highest *Fga* mRNA expression, it was not induced by cold exposure. Posttranscriptional regulation or reduced protein degradation may be considered; however, our finding indicates that the observed increase in FGA protein expression may be attributed to an increased uptake of FGA from the circulation by cold exposure. Our study focused on potential ligand-receptor pairs between BAT and liver that are activated by cold exposure. Since many tissues are affected by cold [57], FGA and/or FGA could be released from tissues other than BAT. In fact, FGA and FN1 were identified as two of the most abundant proteins in the secretome of mice following exercise training [36]. By mapping the organism-wide secretome of exercised mice with 21 cell types and 10 tissues, this study also demonstrated that both proteins are secreted from various tissues, albeit in different proportions, both in the sedentary state and after exercise. FN1 and FGA were secreted at higher levels by BAT than by skeletal muscle, even after exercise, and secretion of FN1 from the liver was > 80% decreased compared to BAT under both conditions [36]. Since BAT secretes a high quantity of these proteins in an exercise/training model, it is highly probable that it secretes an even greater amount when exposed to cold. Given the fact that BAT is the tissue that is most affected and activated by cold [39], the results of this study reinforce our speculation that FN1 and FGA are released from BAT and taken up by the liver in a cardiometabolic state in response to cold stimulation. The upregulation of ITGB1 downstream targets [58, 59] from the liver proteomics datasets provided further evidence for the activation of the interaction between ITGB1 with FGA and ITGB1 with FN1 by cold exposure.

Genetic variations in both genes have also been associated with several cardiometabolic-related phenotypes and traits in humans. The databases of phenotypes used in this study were not limited to those that are directly linked to CMD. Instead, they also included anthropometric, cardiovascular, glycemic, hematologic, lipid, stroke, sleep/discardian, and renal traits, to list just the largest trait groups. Thus, we also found associations with other traits, including blood cell count, venous thromboembolism, and blood pressure. Although the observations were based solely on associations with no indication of direction or mechanism [34], they add further relevance to our findings in mice and the relevance of FGA and FN1 in human CMDs. Fibrinogen, composed of α, β, and γ chains, has traditionally been associated with blood clot formation, but is also linked to cell adhesion and spreading, vasoconstriction and chemotactic activities, and induction of cell division in several cell types [60–62]. However, FGA has never been implicated in CIT and CMDs. FN1 is a major organizer of the extracellular matrix, crucial for various processes such as cell adhesion, shape, migration, and differentiation due to its interactions with multiple receptors [63]. FN1 and its variants have been shown to play a key role during CMDs [64, 65]. Consistent with our findings, increased levels of FN1 in the secretory proteome of brown adipocytes in response to cAMP-mediated thermogenic activation indicate a potential role of this protein as batokine beyond CMDs [9]. Moreover, the combined action of fibrinogen and FN1 promoted liver function and maturation by activating the Wnt/β-catenin pathway via integrins in hepatocytes [64, 66].

The significant but only slight increase in circulating FGA levels of chow-diet fed LdlrKO mice by cold exposure might indicate an association between FGA and the burden of cardiometabolic disease, consistent with hepatic FGA protein abundance being increased upon LDLR deficiency and dietary challenge. In our study, we analyzed networks of regulatory interactions in BAT and liver and identified FGA and FN1 as potential crosstalk factors upon cold exposure. However, further studies are needed to confirm whether the secretion of FN1 (or FGA) by brown adipocytes goes beyond paracrine effects and effectively translates into a release from BAT into circulation. Future experiments using conditional deletion of FGA and/or FN1 in BAT, followed by assessing FGA and FN1 levels in the circulation and the liver, will be instrumental to definitely delineate the precise contributions of BAT-secreted FGA and FN1 to liver function. In addition, further studies should examine the mechanisms of crosstalk between metabolic tissues and the heart and vasculature, which may be particularly interesting in this mouse model.

## Conclusions

In conclusion, this study delved into the intricate changes at the molecular level that occur in response to CIT of a CMD mouse model, shedding light on potential regulatory interactions and crosstalk between BAT and liver. These findings pave the way for future studies exploring the molecular mediators and signaling pathways involved in BAT activation and its impact on cardiometabolic health.

### Electronic supplementary material

Supplementary Material 1.

Supplementary Material 2.

Supplementary Material 3.

Supplementary Material 4.

Supplementary Material 5.

Supplementary Material 6.

## Data Availability

The mass spectrometry proteomics datasets supporting the conclusions of this article have been deposited to the ProteomeXchange Consortium via the PRIDE repository with the dataset identifier PXD051640.

## Abbreviations

BAT: Brown adipose tissue
CMD: Cardiometabolic diseases
CIT: Cold-induced thermogenesis
eWAT: Epididymal white adipose tissue
FA: Fatty acids
FGA: Fibrinogen alpha chain
FN1: Fibronectin 1
HFSCD: High-fat, high-sucrose, high-cholesterol diet
H&E: Hematoxylin and eosin
IPA: Ingenuity pathway analysis
LdlrKO: LDL receptor-deficient
ORO: Oil red O
sWAT: Subcutaneous white adipose tissue
TFs: Transcription factors
TG: Triglyceride
WAT: White adipose tissue
