# Supplementary material for: Identification of regulatory networks and crosstalk factors in brown adipose tissue and liver of a cold-exposed cardiometabolic mouse model
Source: Cardiovasc Diabetol. 2024 Aug 14;23:298. doi: 10.1186/s12933-024-02397-7 (PMC11325583; doi:10.1186/s12933-024-02397-7)
Supplement: Supplementary file 6 — Supplementary Material 6. [file 12933_2024_2397_MOESM6_ESM.pptx]

## Slide 1
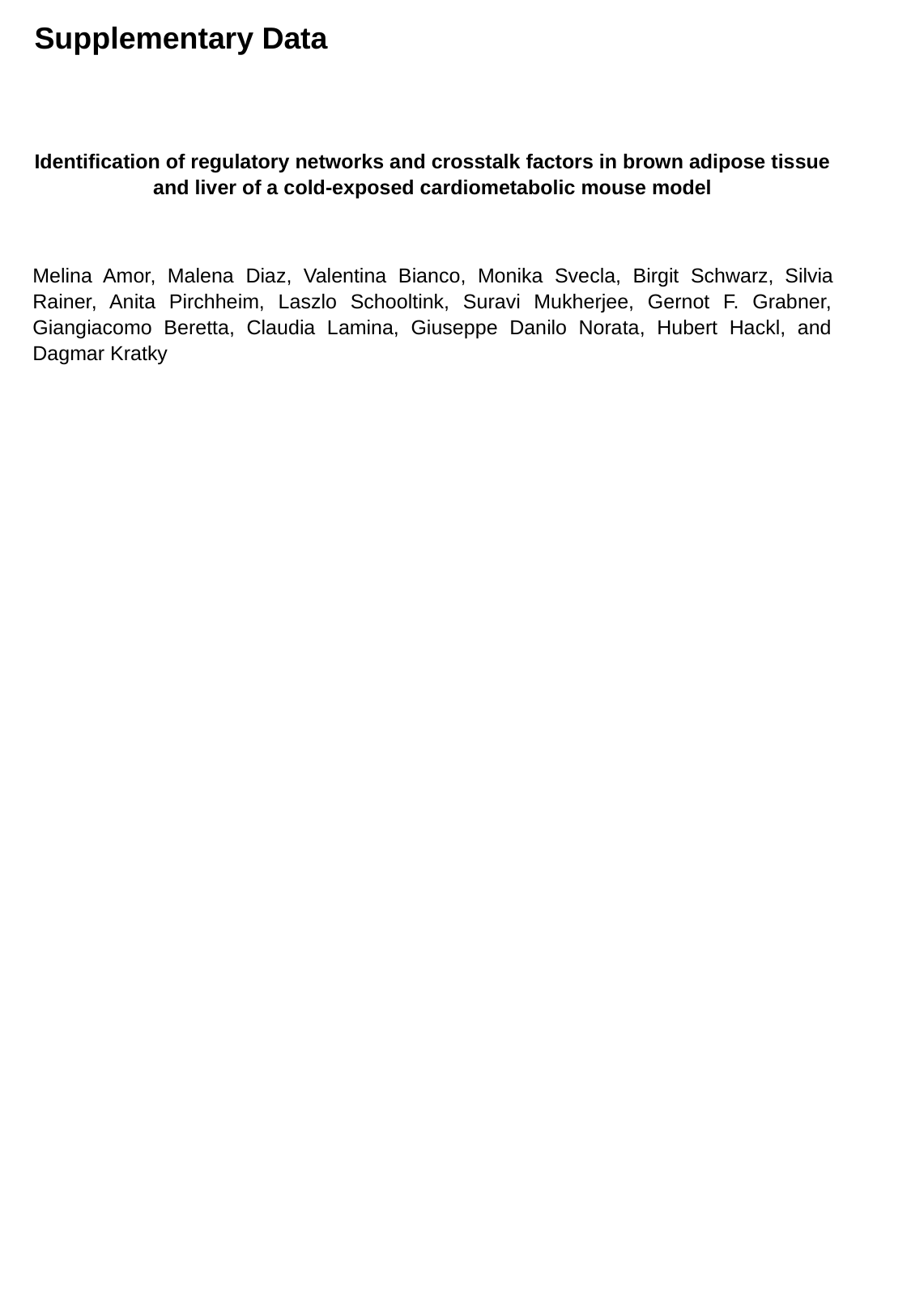

Supplementary Data
Identification of regulatory networks and crosstalk factors in brown adipose tissue and liver of a cold-exposed cardiometabolic mouse model
Melina Amor, Malena Diaz, Valentina Bianco, Monika Svecla, Birgit Schwarz, Silvia Rainer, Anita Pirchheim, Laszlo Schooltink, Suravi Mukherjee, Gernot F. Grabner, Giangiacomo Beretta, Claudia Lamina, Giuseppe Danilo Norata, Hubert Hackl, and Dagmar Kratky

## Slide 2
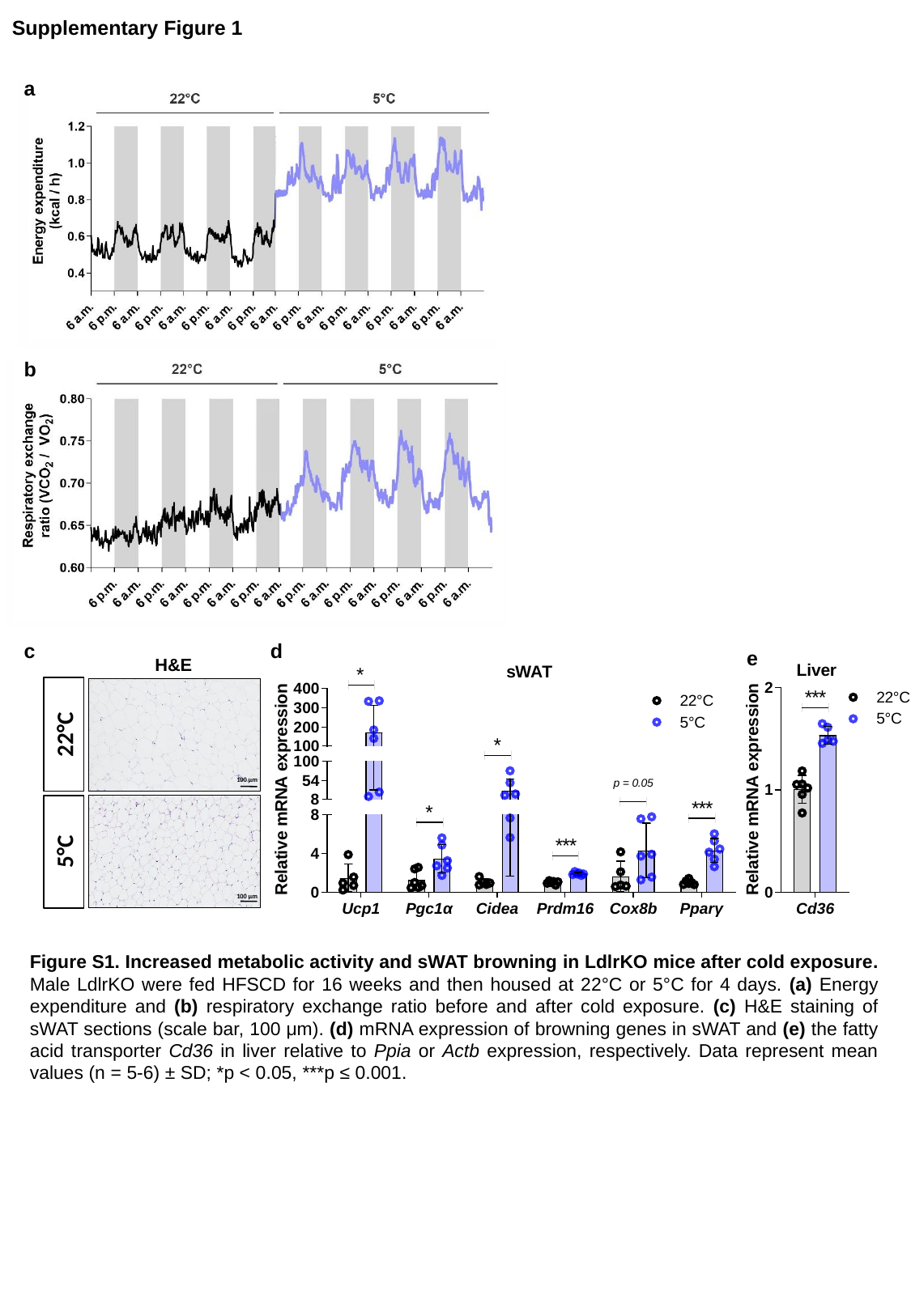

Supplementary Figure 1
a
b
c
d
e
H&E
100 µm
22°C
5°C
100 µm
Figure S1. Increased metabolic activity and sWAT browning in LdlrKO mice after cold exposure. Male LdlrKO were fed HFSCD for 16 weeks and then housed at 22°C or 5°C for 4 days. (a) Energy expenditure and (b) respiratory exchange ratio before and after cold exposure. (c) H&E staining of sWAT sections (scale bar, 100 μm). (d) mRNA expression of browning genes in sWAT and (e) the fatty acid transporter Cd36 in liver relative to Ppia or Actb expression, respectively. Data represent mean values (n = 5-6) ± SD; *p < 0.05, ***p ≤ 0.001.

## Slide 3
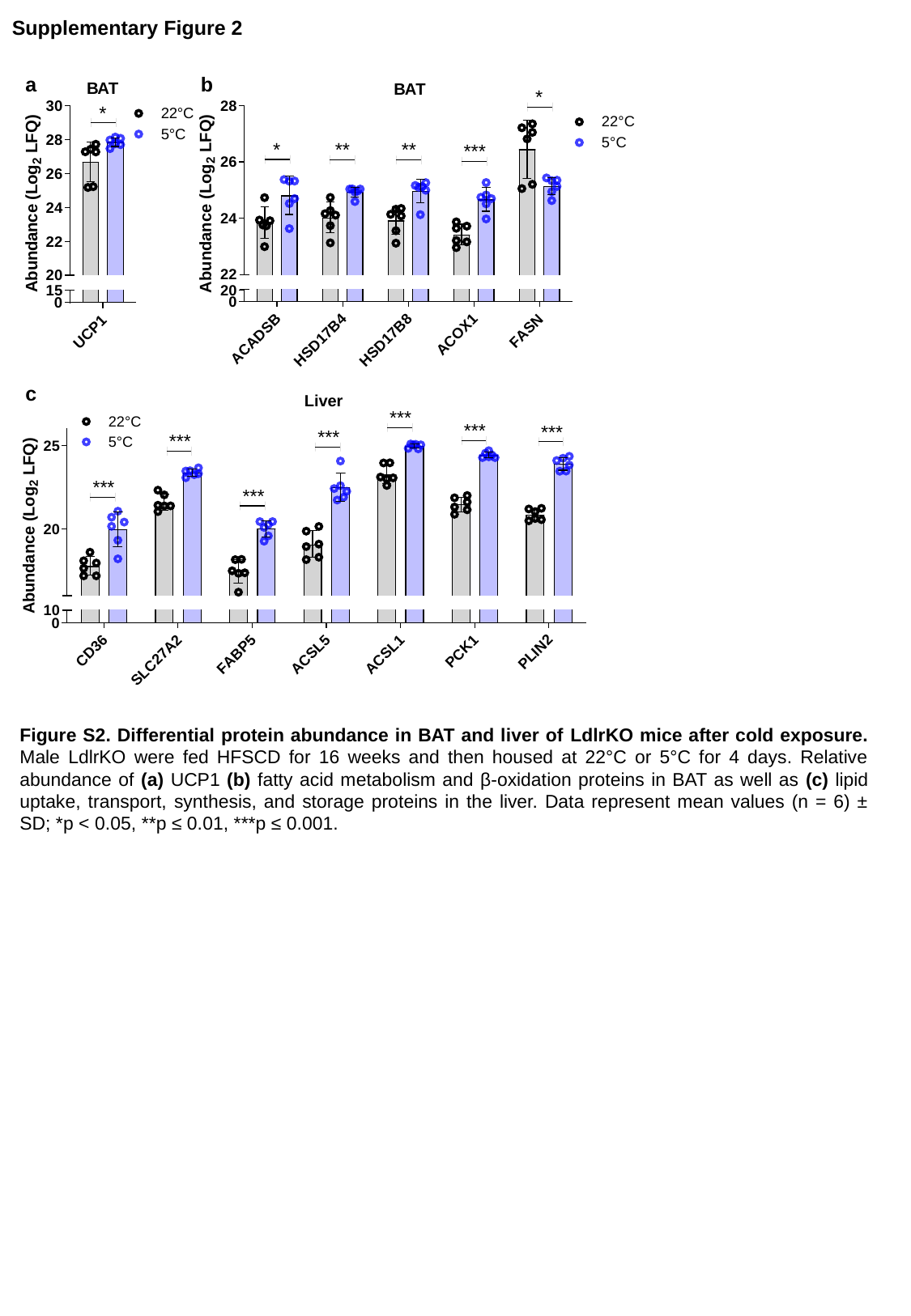

Supplementary Figure 2
a
b
c
Figure S2. Differential protein abundance in BAT and liver of LdlrKO mice after cold exposure. Male LdlrKO were fed HFSCD for 16 weeks and then housed at 22°C or 5°C for 4 days. Relative abundance of (a) UCP1 (b) fatty acid metabolism and β-oxidation proteins in BAT as well as (c) lipid uptake, transport, synthesis, and storage proteins in the liver. Data represent mean values (n = 6) ± SD; *p < 0.05, **p ≤ 0.01, ***p ≤ 0.001.

## Slide 4
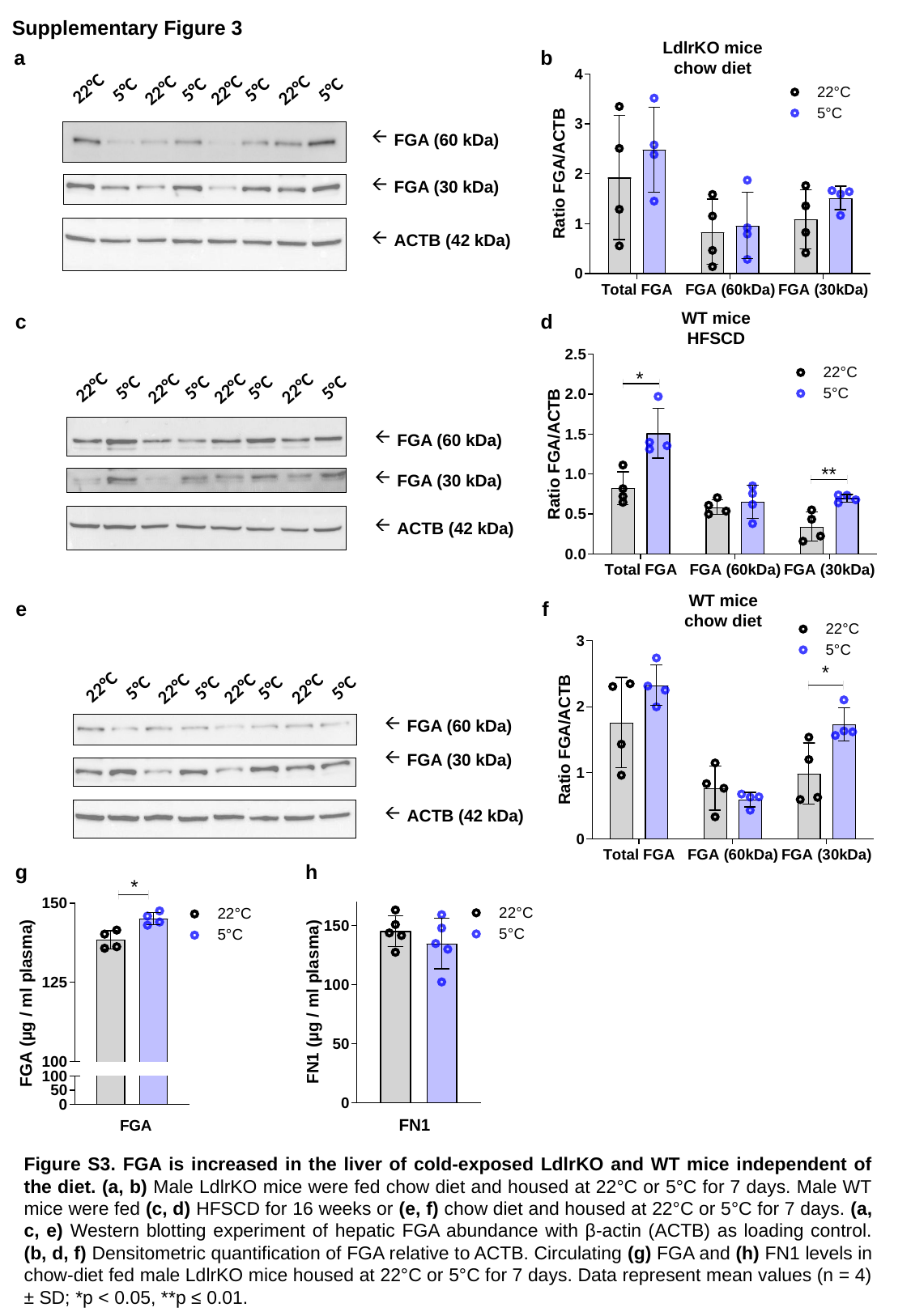

Supplementary Figure 3
LdlrKO mice
chow diet
a
b
22°C
5°C
5°C
5°C
5°C
22°C
22°C
22°C
FGA (60 kDa)
FGA (30 kDa)
ACTB (42 kDa)
WT mice
HFSCD
c
d
22°C
5°C
5°C
5°C
5°C
22°C
22°C
22°C
FGA (60 kDa)
FGA (30 kDa)
ACTB (42 kDa)
WT mice
chow diet
e
f
22°C
5°C
5°C
5°C
5°C
22°C
22°C
22°C
FGA (60 kDa)
FGA (30 kDa)
ACTB (42 kDa)
g
h
Figure S3. FGA is increased in the liver of cold-exposed LdlrKO and WT mice independent of the diet. (a, b) Male LdlrKO mice were fed chow diet and housed at 22°C or 5°C for 7 days. Male WT mice were fed (c, d) HFSCD for 16 weeks or (e, f) chow diet and housed at 22°C or 5°C for 7 days. (a, c, e) Western blotting experiment of hepatic FGA abundance with β-actin (ACTB) as loading control. (b, d, f) Densitometric quantification of FGA relative to ACTB. Circulating (g) FGA and (h) FN1 levels in chow-diet fed male LdlrKO mice housed at 22°C or 5°C for 7 days. Data represent mean values (n = 4) ± SD; *p < 0.05, **p ≤ 0.01.

## Slide 5
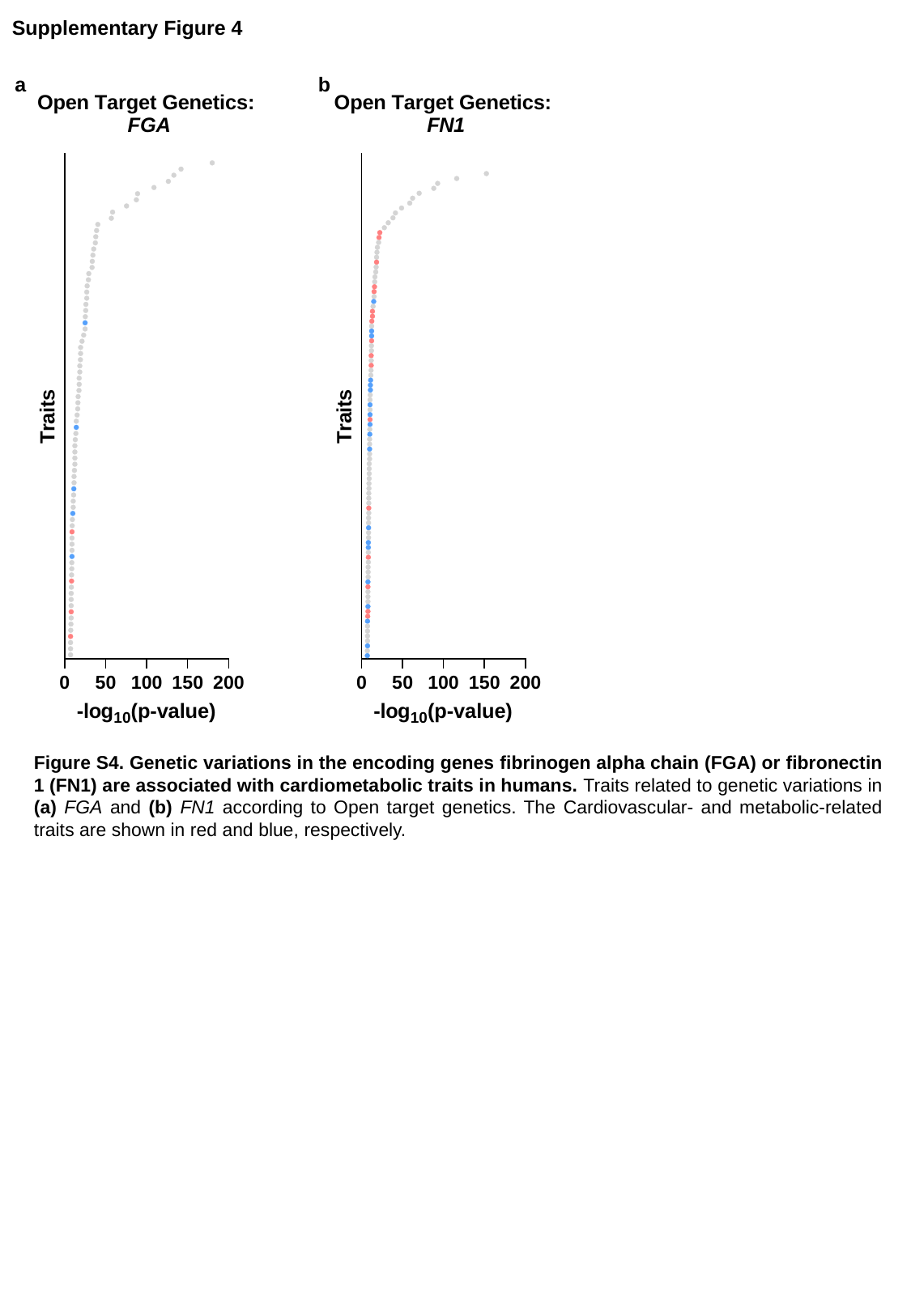

Supplementary Figure 4
a
b
Figure S4. Genetic variations in the encoding genes fibrinogen alpha chain (FGA) or fibronectin 1 (FN1) are associated with cardiometabolic traits in humans. Traits related to genetic variations in (a) FGA and (b) FN1 according to Open target genetics. The Cardiovascular- and metabolic-related traits are shown in red and blue, respectively.

## Slide 6
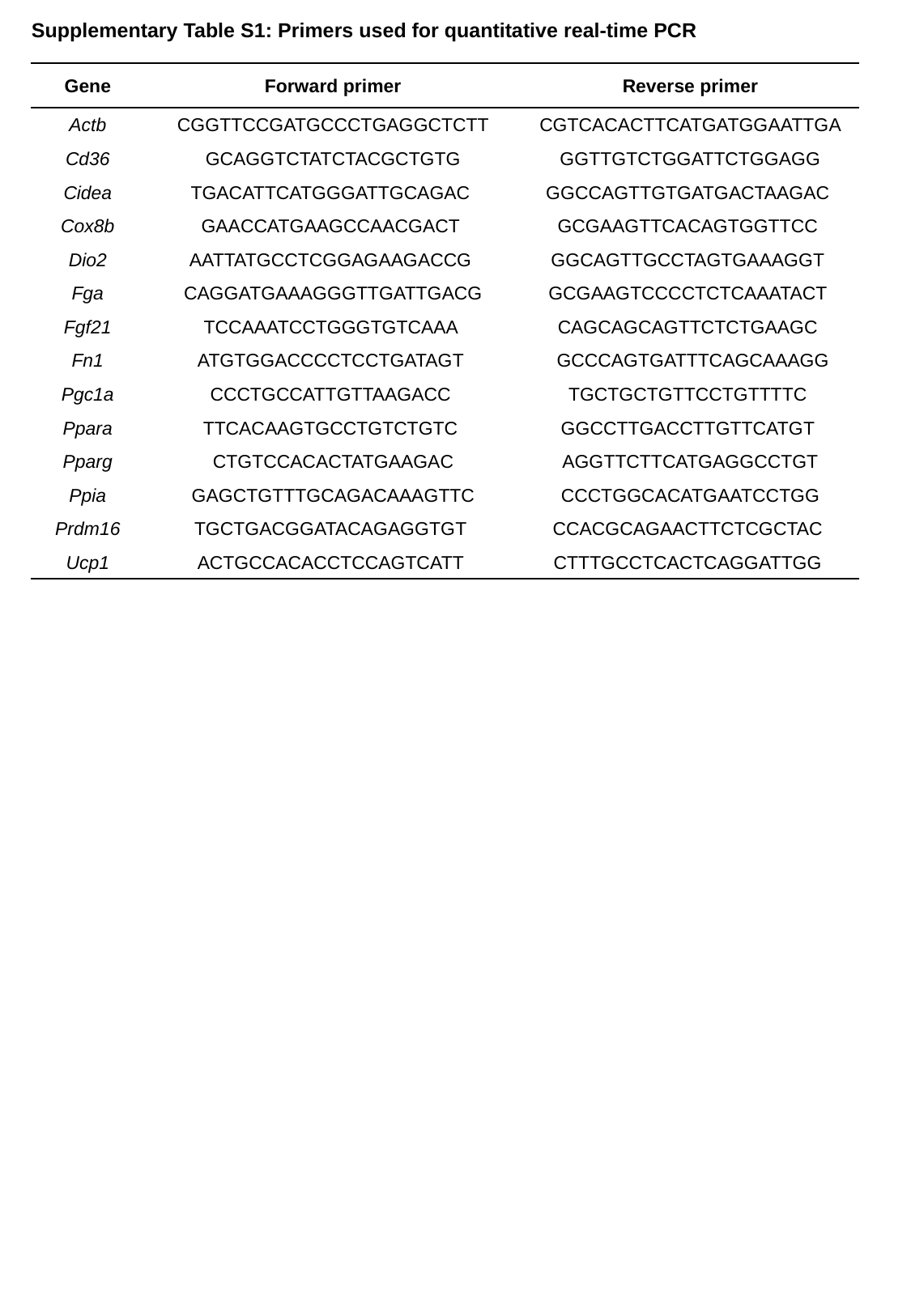

Supplementary Table S1: Primers used for quantitative real-time PCR
| Gene | Forward primer | Reverse primer |
| --- | --- | --- |
| Actb | CGGTTCCGATGCCCTGAGGCTCTT | CGTCACACTTCATGATGGAATTGA |
| Cd36 | GCAGGTCTATCTACGCTGTG | GGTTGTCTGGATTCTGGAGG |
| Cidea | TGACATTCATGGGATTGCAGAC | GGCCAGTTGTGATGACTAAGAC |
| Cox8b | GAACCATGAAGCCAACGACT | GCGAAGTTCACAGTGGTTCC |
| Dio2 | AATTATGCCTCGGAGAAGACCG | GGCAGTTGCCTAGTGAAAGGT |
| Fga | CAGGATGAAAGGGTTGATTGACG | GCGAAGTCCCCTCTCAAATACT |
| Fgf21 | TCCAAATCCTGGGTGTCAAA | CAGCAGCAGTTCTCTGAAGC |
| Fn1 | ATGTGGACCCCTCCTGATAGT | GCCCAGTGATTTCAGCAAAGG |
| Pgc1a | CCCTGCCATTGTTAAGACC | TGCTGCTGTTCCTGTTTTC |
| Ppara | TTCACAAGTGCCTGTCTGTC | GGCCTTGACCTTGTTCATGT |
| Pparg | CTGTCCACACTATGAAGAC | AGGTTCTTCATGAGGCCTGT |
| Ppia | GAGCTGTTTGCAGACAAAGTTC | CCCTGGCACATGAATCCTGG |
| Prdm16 | TGCTGACGGATACAGAGGTGT | CCACGCAGAACTTCTCGCTAC |
| Ucp1 | ACTGCCACACCTCCAGTCATT | CTTTGCCTCACTCAGGATTGG |
